# Superhydrophilic Fluorinated Polymer Probe for Zero-Background ^19^F MRI with Adaptable Targeting Ability

**DOI:** 10.1021/acsami.4c14715

**Published:** 2024-11-15

**Authors:** Chang Guo, Xiaoyao Xiong, Xinxing Zhao, Yumin Li, Sijia Li, Suying Xu, Tony D. James, Leyu Wang

**Affiliations:** † State Key Laboratory of Chemical Resource Engineering, College of Chemistry, Beijing University of Chemical Technology, Beijing 100029, China; ‡ Department of Chemistry, University of Bath, BA2 7AY Bath, United Kingdom; § School of Chemistry and Chemical Engineering, Henan Normal University, Xinxiang 453007, China

**Keywords:** ^19^F magnetic resonance imaging, nanoprobes, superhydrophilic polymer, fluorinated polymer, tumor targeting

## Abstract

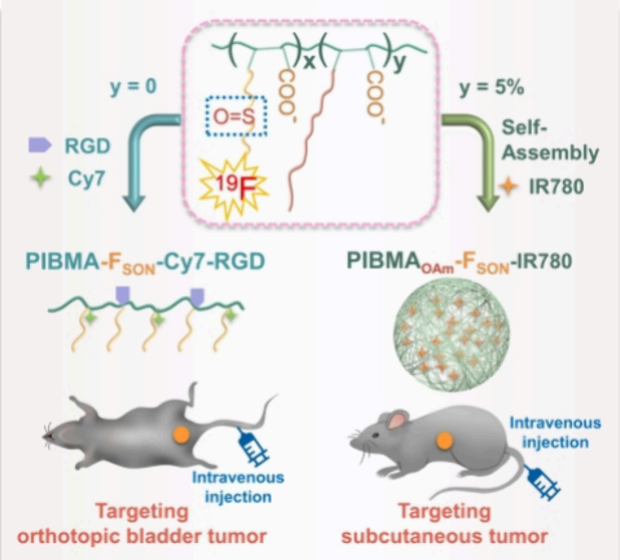

^19^F magnetic resonance imaging (^19^F MRI), with zero background, high tissue penetration depth, excellent spatial resolution, and nonradioactive features, has attracted considerable attention but faces tough challenges due to the shortage of sensitive and selective targetable probes. Herein, we report a biocompatible and highly sensitive ^19^F MRI probe with an adaptable tumor-targeting ability. The fluorine-grafted polymer (PIBMA-F_SON_) probes were rich with sulfoxide and carboxy groups, containing a high fluorine content (∼17 wt %). The probes exhibit superhydrophilicity, strong ^19^F MRI signals (enhancement of ∼95-fold), long transverse relaxation time (*T*_2_, 422 ms), and excellent ^19^F MRI capability. Conjugation using a targeting peptide (Arg-Gly-Asp, RGD) afforded ultrasmall soft polymer probes (PIBMA-F_SON_-RGD) with superhydrophilicity and tumor-targeting ability suitable for the ^19^F MRI of orthotopic bladder cancer. Amidification of 5% of the carboxylate units with oleylamine resulted in PIBMA_OAm_-F_SON_ nanoprobes (NPs) via self-assembly, displaying different targeting toward subcutaneous tumors. Further grafting with near-infrared (NIR) dyes renders the probe suitable for NIR-fluorescence and ^19^F MRI dual-modality imaging. This study provides a suitable approach for designing highly sensitive and zero-background ^19^F MRI probes with a tunable tumor-targeting ability.

## Introduction

Novel probes for the sensitive and selective imaging of tissues of interest with high penetration depth and low background are highly desirable in biomedical fields, yet many challenges remain, despite significant progress having been made in the probe fabrication for imaging including near-infrared (NIR) luminescence imaging, photoacoustic imaging, positron emission tomography (PET), computed tomography (CT), and magnetic resonance imaging (MRI).^1−4^^1^H MRI, as one of the most promising noninvasive imaging techniques, is widely used for clinical diagnosis owing to its excellent spatial resolution, unlimited tissue penetration depth, and nonradioactive nature.^5−8^^19^F MRI retains the advantages of ^1^H MRI, while exhibiting almost zero background, resulting in quantitative analysis and providing a “hot spot” for areas of interest.^9−17^

Unlike ^1^H MRI which uses relaxation time-weighted imaging, ^19^F MRI is a spin density-weighted imaging technique, where the signal is proportional to the content of fluorine atoms possessing the same magnetic equivalence. In order to obtain highly sensitive ^19^F MRI, it is important to increase the number of fluorine atoms in the MRI probes. However, increasing the number of fluorine atoms can result in a higher restriction of molecular mobility, which, in turn, causes the shortening of transverse relaxation time (*T*_2_) and thus attenuation of the ^19^F MRI signal.^18^ Inorganic nanomaterials such as CaF_2_ nanocrystals exhibit significant potential for in vivo ^19^F MRI,^19,20^ where a specific ultrashort echo time (UTE) sequence could be used to overcome the ultrashort *T*_2_ value. Alternatively, perfluorocarbon (PFC) nanoemulsion consisting of a PFC core coated by a lipid shell is one of the most studied ^19^F MRI nanoprobes (NPs) for preclinical studies.^21−23^ However, the poor stability and health concerns associated with long retention times in the circulating blood have encouraged researchers to explore other sensitive and biocompatible ^19^F MRI probes.

Polymeric ^19^F MRI probes exhibit superior performance in terms of stability, biocompatibility, and structural versatility.^24−28^ Nevertheless, due to the hydrophobic properties of C–F bonds, an increase in fluorine atom content is often compromised by hydrophobic aggregation-induced signal attenuation.^29^ As such, a key challenge lies in how to increase the fluorine content and still maintain a suitable relaxation time (long *T*_2_). Over the past decade, significant research has been devoted to the structure design of polymers capable of improving ^19^F NMR properties. Thurecht et al.^30^ designed a hyperbranched polymer, which utilized a branched polymeric structure coupled with random incorporation of trifluoroethyl groups within a hydrophilic PEG-based macrostructure to maintain sufficient segmental mobility. Nevertheless, these polymers have a ^19^F content of <5 wt %. Moonshi et al.^31^ increased the fluorine content (∼10 wt %) using a perfluoropolyether (PFCE) based hyperbranched polymer, where PFCE provides a strong ^19^F MRI signal and hydrophilic oligo (ethylene glycol) methyl ether acrylate enhances the aqueous solubility. In addition, recent studies have focused on the fabrication of hydrophilic fluorinated monomers to improve the relaxation time of fluorinated probes,^32,33^ which indicated that the local environment of the fluorine atoms played a vital role in the performance of ^19^F MRI probes.

Besides the strong ^19^F MRI signal, with respect to in vivo imaging of the tumor, the imaging quality is also closely related to the specific targeting ability of the probes. As such, appropriate targeting moieties such as peptides, aptamers, and sugar residues have been used to increase the targeting efficiency.^34^ Apart from active targeting, the sizes of the probes endow additional targeting ability via the enhanced permeability and retention effect (passive targeting). However, ^19^F NMR is sensitive to changes of the surrounding environment and the assembly of probes can induce variation in relaxation time in addition to enhancing the targeting ability. Therefore, it becomes challenging to use passive targeting and still maintain the ^19^F MRI performance of the probes.

**Scheme 1:**
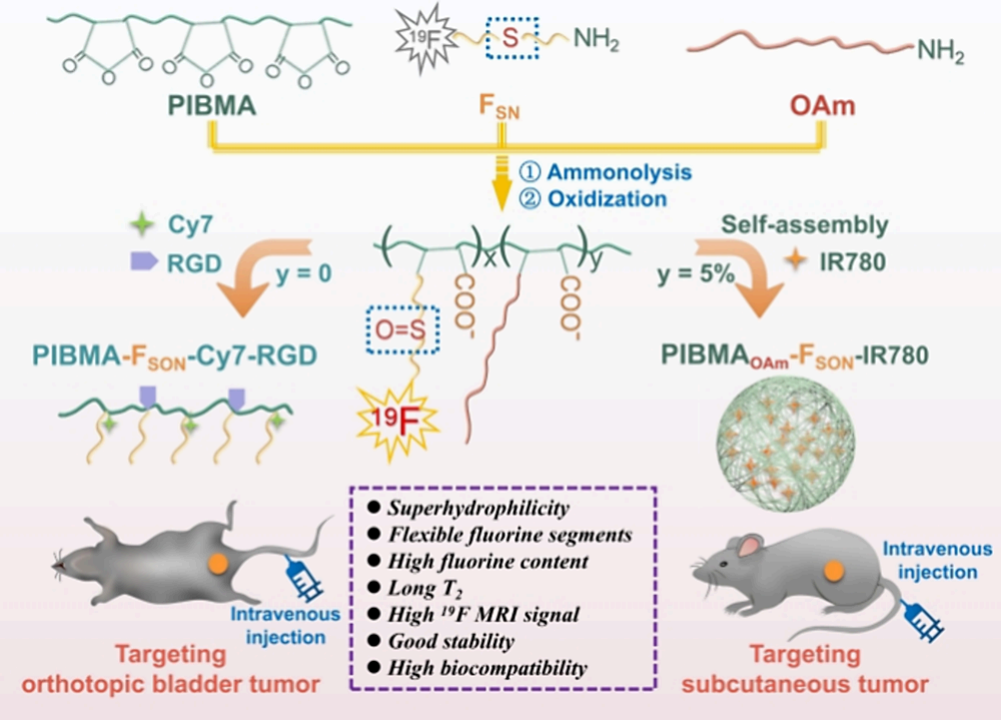
Schematic Illustration of Fabrication of Superhydrophilic Fluorinated Polymer Probes for Selective Tumor Targeting and Imaging

Herein, we designed a highly sensitive ^19^F MRI probe based on a superhydrophilic fluorine-grafted polymer enriched with sulfoxide and carboxy groups (Scheme 1), which exhibited different targeting specificities based on regulation of the assembly behavior. Specifically, poly(isobutylene-maleic anhydride) (PIBMA) was modified with 2-(trifluoroethyl) thioethylamine (F_SN_) to obtain PIBMA-F_SN_ using a ring-opening reaction (Figures S1–S7). The sulfur ether group in PIBMA-F_SN_ was further oxidized to the sulfoxide group, affording a superhydrophilic fluorinated polymer (PIBMA-F_SON_). The PIBMA-F_SON_ polymer exhibited high fluorine content (∼17 wt %) and long *T*_2_ value (422 ms) and as such is a promising candidate as a ^19^F MRI probe. Significantly, the free carboxy group on the polymer could be readily conjugated using a targeting peptide (Arg-Gly-Asp, RGD) and a near-infrared (NIR) fluorescent dye (heptamethine cyanine dye derivatives, Cy7-amine) for targeted dual-modality bladder cancer imaging via intravenous injection of these ultrasmall soft polymer probes. Moreover, when 5% of the carboxylate units were amidated by oleylamine, self-assembled PIBMA_OAm_-F_SON_-IR780 nanoprobes (NPs) with a long *T*_2_ value (437 ms), which could simultaneously encapsulate the NIR dye (IR780), were obtained. The nearly unchanged *T*_2_ value implies that the self-assembly of the polymer hardly influenced the relaxation of the ^19^F nuclei and thus the ^19^F MRI properties. In addition, PIBMA_OAm_-F_SON_-IR780 NPs exhibited selective targeting capability toward subcutaneous tumors, and vastly different biodistribution when compared to the PIBMA-F_SON_ polymer. This study provides a facile method for developing highly sensitive superhydrophilic ^19^F MRI probes with controllable targeting behavior and provides a novel approach for preparing a multifunctional imaging platform.

## Results and Discussion

### Synthesis of the PIBMA-F_SON_

**Figure 1:**
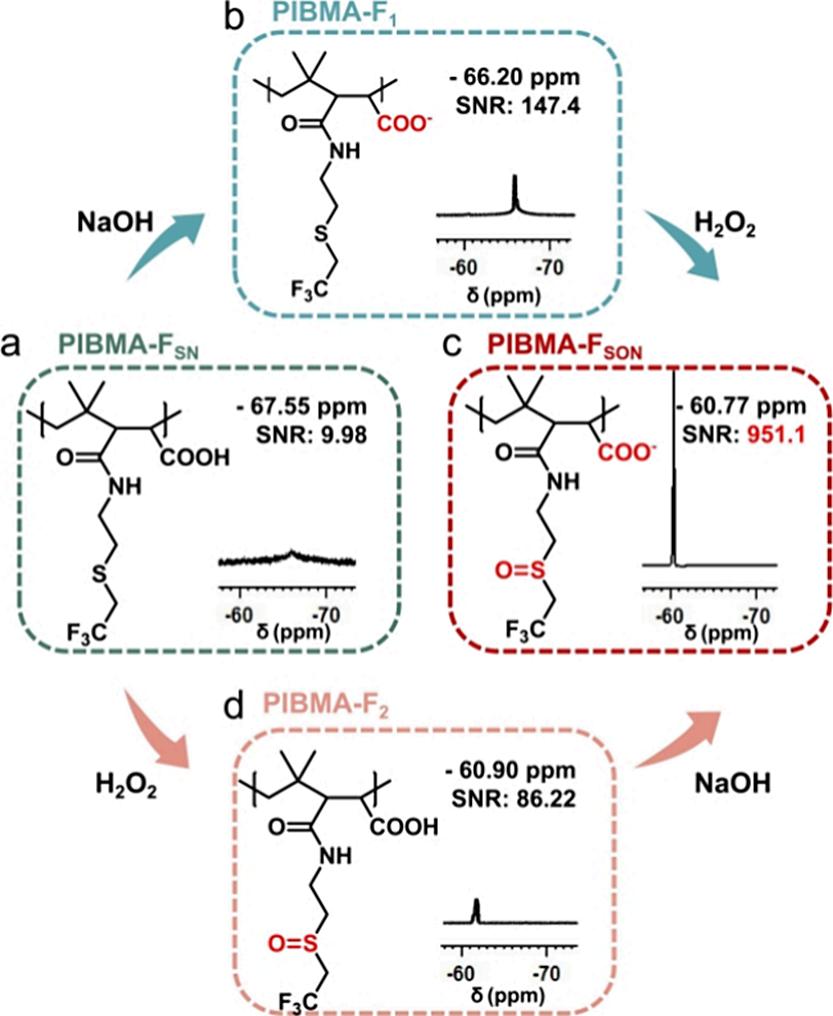
Chemical structure and ^19^F NMR spectrum of (a) PIBMA-F_SN_, (b) PIBMA-F_1_, (c) PIBMA-F_SON_, and (d) PIBMA-F_2_ in water (20 mg/mL).

As shown in Figure 1a, the ^19^F NMR spectrum of PIBMA-F_SN_ exhibited a single peak at −67.55 ppm with a broad half peak width (peak width) of ∼2467.60 Hz. By adjustment of the pH to slightly alkaline conditions, nanometer-sized particles (PIBMA-F_1_) were obtained (Figure 1b). The transmission electron microscopy (TEM) images (Figure S8) and dynamic light scattering (DLS) analysis (Figure S9) indicate that PIBMA-F_1_ was a nanosphere with an average diameter of 80 ± 22 nm. The chemical shift exhibited a slight downfield movement to −66.20 ppm (Figure 1b), and the ^19^F NMR signal-to-noise ratio (SNR) increased ∼15-fold, but was still insufficient for ^19^F MRI applications. Such results indicated that the aggregation of fluorine-containing moieties due to the hydrophobic interaction severely impaired the ^19^F NMR intensities. Finally, hydrogen peroxide (H_2_O_2_) was used to oxidize the hydrophobic thioether group to the hydrophilic sulfoxide group to improve the hydrophilicity of the polymer. The conversion of PIBMA-F_SN_ into PIBMA-F_SON_ was completed in 60 h (Figure S10). The successful transformation of thioether groups into sulfoxide groups was verified using ^1^H NMR (Figure S11) and Fourier transform infrared (FTIR) spectroscopy (Figure S12), where the sulfoxide S=O stretching vibration at 1026 cm^–1^ was observed for the resulting PIBMA-F_SON_.^35^ As shown in Figure 1c, the ^19^F NMR spectrum of PIBMA-F_SON_ exhibited a single peak at −60.77 ppm with a Peakw of ∼75.29 Hz, which was narrow enough for ^19^F MRIs. Due to the superhydrophilicity of the sulfoxide and carboxylate groups, the PIBMA-F_SON_ polymer can be directly dissolved in aqueous solution (Figures S13 and S14), achieving a total ^19^F NMR SNR enhancement of ∼95-fold. However, through only the oxidation of PIBMA-F_SN_ (PIBMA-F_2_), it was difficult to obtain a good ^19^F NMR SNR. As shown in Figure 1d, the ^19^F NMR SNR of PIBMA-F_2_ increased ∼9-fold. Therefore, both the sulfoxide and anionic carboxylate groups are required for satisfactory ^19^F NMR signal enhancement, and therefore PIBMA-F_SON_ was used in all subsequent evaluations.

### Characterization of the PIBMA-F_SON_

**Figure 2:**
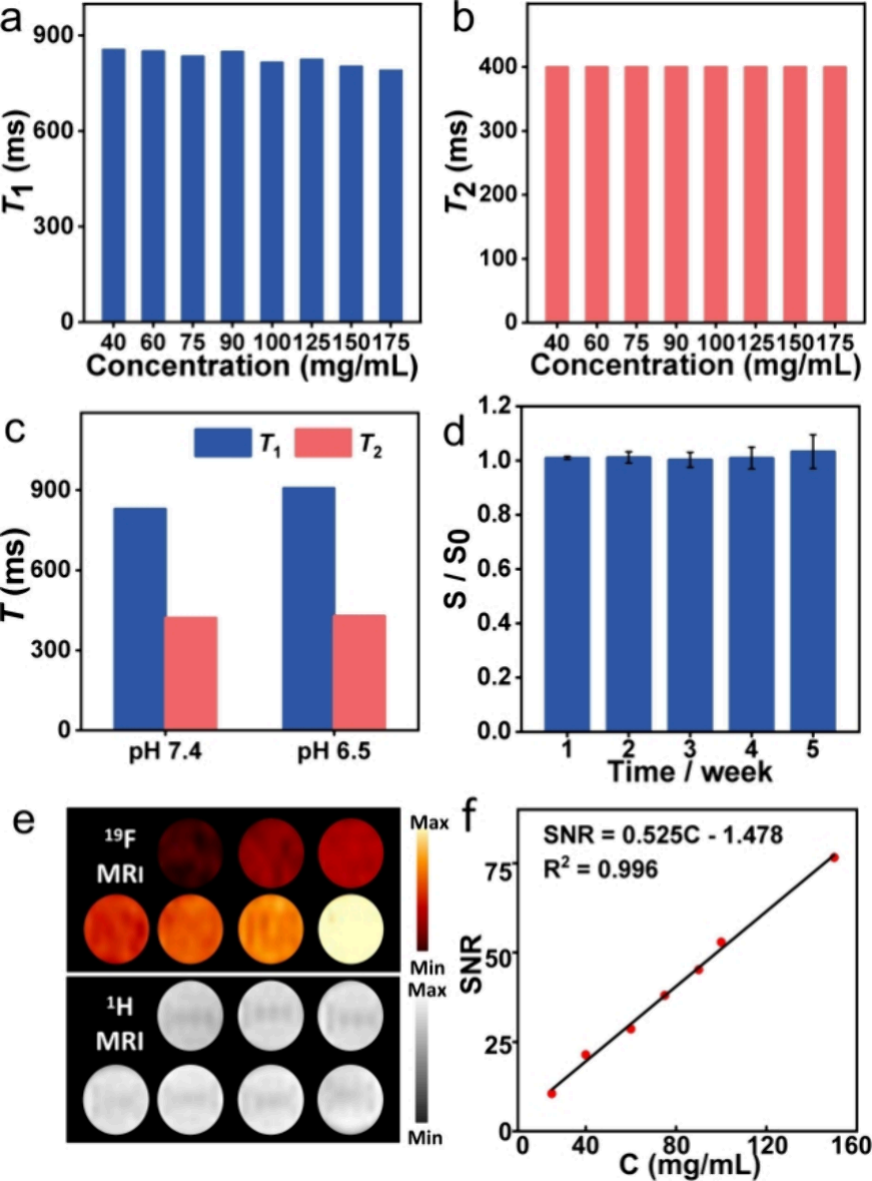
(a) *T*_1_ and (b) *T*_2_ values of PIBMA-F_SON_ with different concentrations. (c) Comparison of *T*_1_ and *T*_2_ of PIBMA-F_SON_ at different pH conditions. (d) Evolution of the ^19^F NMR SNR of PIBMA-F_SON_ along with time. *S* and *S*_0_ refer to the ^19^F SNR of the probe (PBS buffer) at the day of the test and the initial state, respectively. (e) ^19^F (top) and ^1^H (bottom) MR images of PIBMA-F_SON_ with various concentrations. (f) Linear plot of ^19^F MRI SNR versus PIBMA-F_SON_ concentration.

PIBMA-F_SON_ exhibited excellent relaxation performance, as evidenced by the longitudinal relaxation time (*T*_1_) and transverse relaxation time (*T*_2_) values shown in Figure 2a,b. In addition, it was found that no significant variations of *T*_1_ and *T*_2_ were observed over a concentration range from 40 to 175 mg/mL, implying that fluorine atoms on the polymer retain excellent relaxation properties. Also, the ^19^F NMR performance was maintained at pH 7.4 and 6.5 (Figure 2c) since the carboxylate group exists mainly in its anionic form under such pH conditions (Figure S15). In addition, the probes exhibited excellent stability over 28 days (Figure 2d) without observable precipitation (Figure S16). PIBMA-F_SON_ has a fluorine content of ∼17 wt % (Figure S17), suggestive of the excellent ^19^F MRI potential. As suggested in Figure 2e, a series of PIBMA-F_SON_ solutions with various concentrations were used for ^19^F MRI, which indicated that the ^19^F MRI SNR increased in proportion to the concentration of PIBMA-F_SON_ (Figure 2f). This linear relationship indicates no aggregation of fluorine atoms and attenuation of the ^19^F MRI over high concentrations of up to 150 mg/mL. Furthermore, over 80% cell viability was observed (Figure S18) for PIBMA-F_SON_ with concentrations up to 2 mg/mL after incubation with MB49 cells (bladder cancer cell) and MREpiC cells (normal cell) for 48 h, respectively. The excellent biocompatibility was also confirmed using the degree of erythrocyte hemolysis, where no apparent hemolysis was observed even at high concentrations (75 mg/mL) of PIBMA-F_SON_ (Figure S19a) with good maintenance of red blood cell numbers (Figure S19b).

### ^19^F MRI of the PIBMA-F_SON_ In Vivo

**Figure 3:**
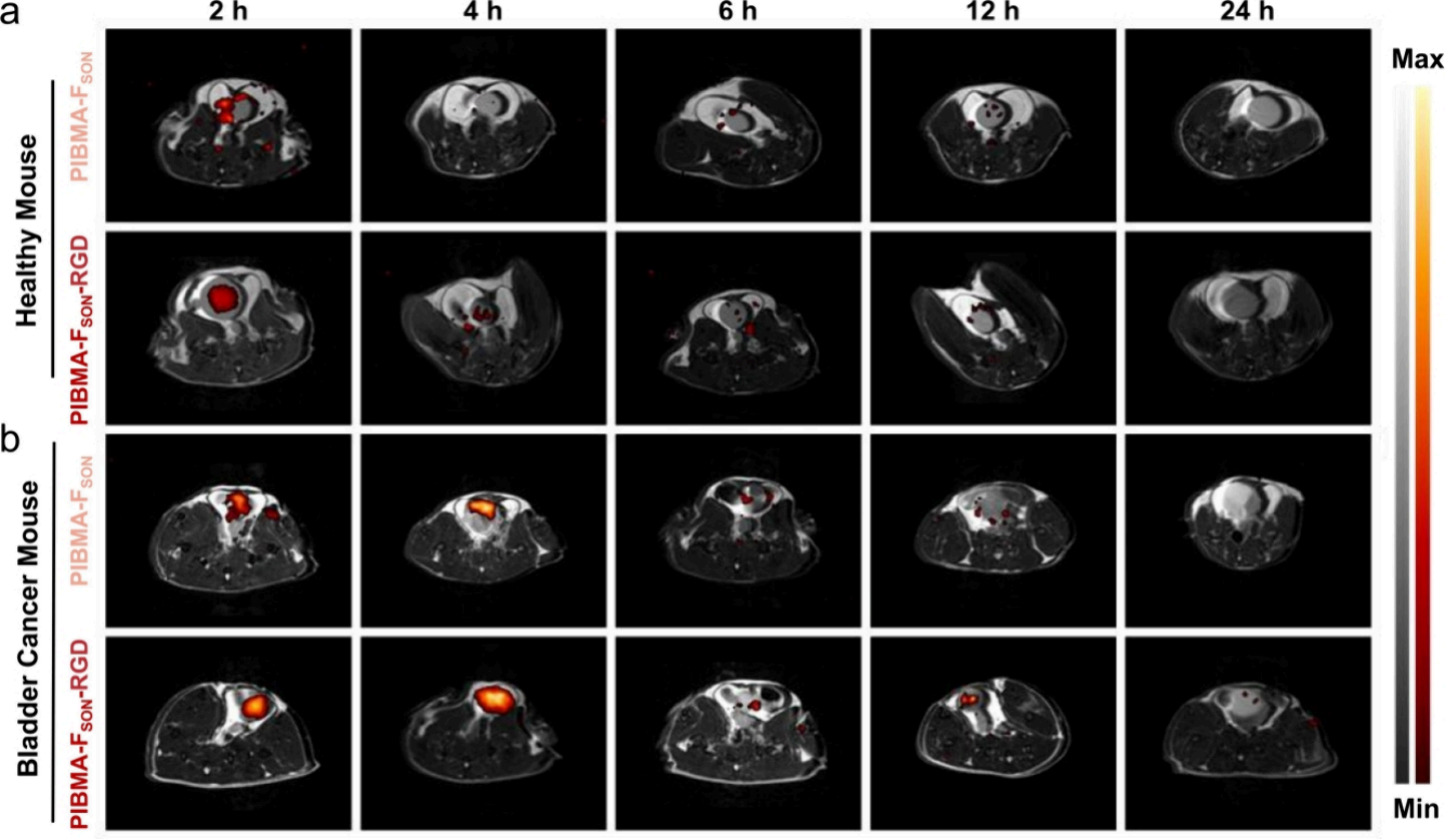
In vivo ^1^H (black-and-white) and ^19^F (colorful) MRI overlaid images of (a) healthy mouse and (b) orthotopic bladder cancer model in the C57BL/6 mouse post intravenous injection (i.v.) with PIBMA-F_SON_ and PIBMA-F_SON_-RGD at different time intervals.

Encouraged by the excellent biocompatibility, stability, and satisfactory ^19^F MRI properties, we carried out the in vivo ^19^F MRI for orthotopic bladder tumors. Bladder cancer, as one of the most common malignancies of the urinary system, usually originates from the epithelial lining of a urinary bladder.^36−38^ Critical issues in bladder cancer management are the local recurrence of disease and accurate delineation of tumor margins intraoperatively. Currently, cystoscopy is still the standard method for patients with urinary symptoms,^39^ which often results in discomfort for patients due to the invasive nature of cystoscopy. Therefore, it is important to develop effective noninvasive imaging technology. Therefore, these PIBMA-F_SON_ polymers were explored for in vivo imaging of a mouse bearing orthotopic bladder cancer *via* intravenous injection. As can be seen from Figure 3a, for the healthy mouse, PIBMA-F_SON_ was metabolized rapidly within about 4 h. As a comparison, PIBMA-F_SON_-RGD, which was prepared by conjugating PIBMA-F_SON_ with a short targeting peptide, displayed significant ^19^F MRI signal enhancement as well as long retention at tumor sites (Figure 3b). The probe remained attached to the tumor site even after the urine was voided. The specificity of PIBMA-F_SON_-RGD toward tumor sites was further verified at a cellular level. Both PIBMA-F_SON_-RGD and PIBMA-F_SON_ were labeled with 5-aminofluorescein for fluorescence imaging. As shown in Figure S20 (Supporting Information), the group with PIBMA-F_SON_-RGD displayed more intense fluorescence than that of the PIBMA-F_SON_ group after incubation for 2 h, implying the specific labeling of MB49 cells with PIBMA-F_SON_-RGD.

Taking advantage of the large number of carboxylate groups on PIBMA-F_SON_, Cy7-amine dye could be covalently attached to the polymer to form PIBMA-F_SON_-Cy7 and PIBMA-F_SON_-Cy7-RGD, which displayed intense emission with NIR-fluorescence imaging capability (Figure S21). As can be seen from Figure S22, the mice bearing bladder cancer tumors displayed a much brighter NIR fluorescence image after being treated with PIBMA-F_SON_-Cy7-RGD via tail vein injection. However, for the control group without RGD, the fluorescence of the tumor site was much weaker, which was consistent with the observations using ^19^F MRI. In addition, blood samples and major organs of the mice were harvested for serological (Figure S23) and histological analysis (Figure S24), 7 days post intravenous injection of PIBMA-F_SON_. The major organs (heart, liver, spleen, lung, kidney, and bladder) of tumor-bearing mice injected with PIBMA-F_SON_ and PIBMA-F_SON_-RGD exhibited no noticeable histological changes compared with those of healthy mice, suggesting the excellent biocompatibility and facile clearance of these polymer probes.

### Synthesis and Characterization of the PIBMA_OAm_-F_SON_

**Figure 4:**
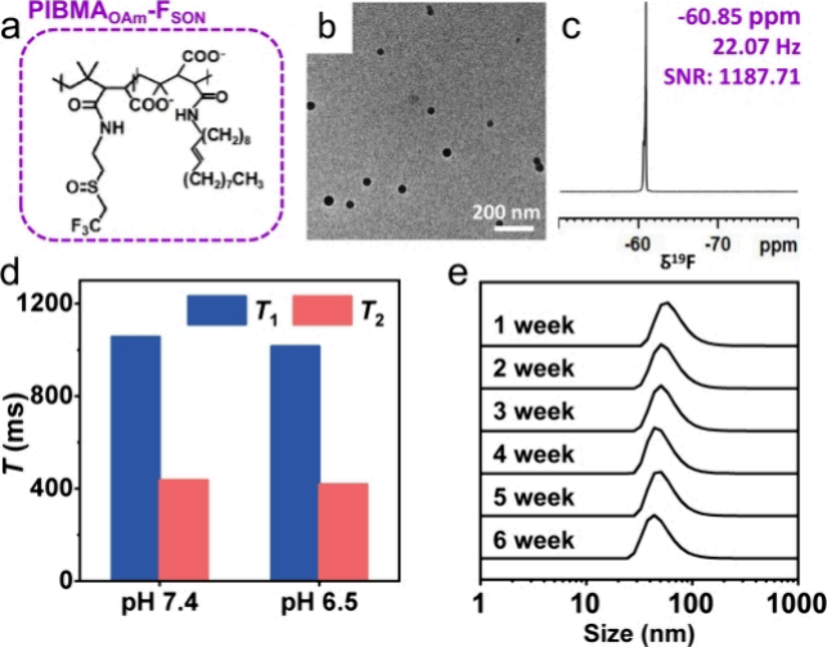
(a) Chemical structures of PIBMA_OAm_-F_SON_. TEM image (b) and ^19^F NMR spectrum (c) of PIBMA_OAm_-F_SON_-IR780 NPs. (d) Comparison of *T*_1_ and *T*_2_ of PIBMA_OAm_-F_SON_-IR780 NPs at different pH values. (e) Evolution of particle size along with incubation time. Inset photographs were PIBMA_OAm_-F_SON_-IR780 NPs powder and PIBMA_OAm_-F_SON_-IR780 NPs redispersed in PBS.

When 5% of the carboxylate units of the polymer were amidated using oleylamine, amphiphilic PIBMA_OAm_-F_SON_ (Figure 4a) was obtained and characterized by ^1^H NMR and FTIR (Figures S25 and S26). On the basis of the hydrophobic interaction between long alkyl chains, amphiphilic PIBMA_OAm_-F_SON_ enables self-assembly into spherical nanostructures. In addition, IR780 (hydrophobic NIR dye) could be encapsulated in the hydrophobic core of PIBMA_OAm_-F_SON_ during the fabrication process, to generate functional PIBMA_OAm_-F_SON_-IR780 NPs (Figure 4b) with a DLS size of 67 ± 25 nm (Figure S27) under optimized conditions (Figures S28–S30). PIBMA_OAm_-F_SON_-IR780 NPs exhibited intense emission with an NIR-fluorescence imaging capability (Figure S31). Despite self-assembly, PIBMA_OAm_-F_SON_-IR780 NPs exhibited a sharp ^19^F NMR peak at around −60.85 ppm with a Peakw of ∼22.07 Hz (Figure 4c), suggesting that the fluorinated group on the PIBMA_OAm_-F_SON_-IR780 NPs displayed favorable mobility, which could be attributed to the hydrophilic sulfoxide nearby. Similarly, the ^19^F NMR signal intensity displayed typical concentration-dependent features (Figure S32) with stable *T*_1_ and *T*_2_ values at both pH 7.4 and 6.5 (Figure 4d). Of particular note is the long *T*_2_ (437 ms). Such a long *T*_2_ is not typically observed for fluorinated polymeric nanoparticles due to aggregation of the fluorinated groups in polymers with high fluorine content. These results indicated that the fluorine atoms on the polymeric nanoparticles maintain excellent relaxation properties.

Moreover, PIBMA_OAm_-F_SON_-IR780 NPs, with a zeta potential of −36.3 mV (Figure S33), were stable in an aqueous solution for a period of over 6 weeks, and neither aggregation nor ^19^F NMR signal loss was observed (Figures 4e and S34). Lyophilization to generate a powder and redissolution does not affect the morphology and ^19^F NMR signals of PIBMA_OAm_-F_SON_-IR780 NPs (Figures S35 and S36), confirming the great potential for practical applications.

### ^19^F MRI of the PIBMA_OAm_-F_SON_ In Vitro and In Vivo

**Figure 5:**
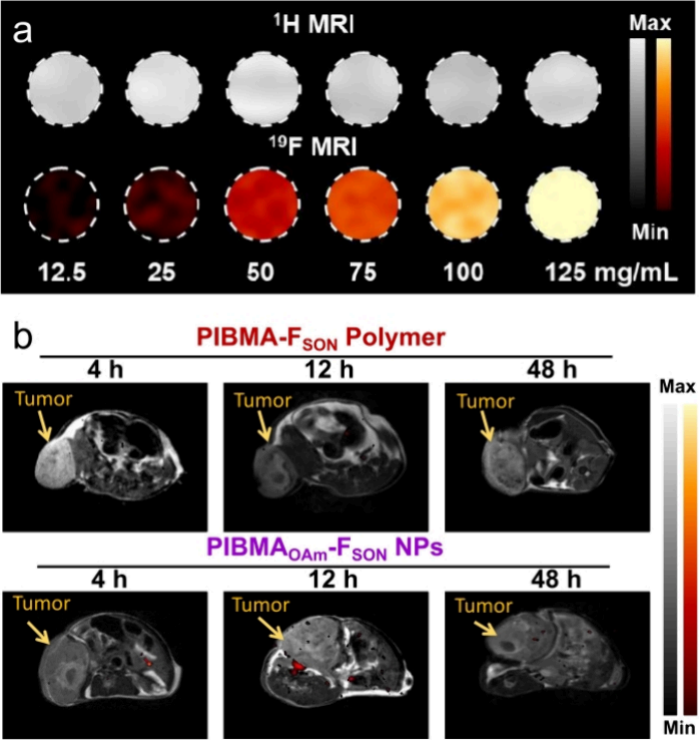
(a) ^1^H and ^19^F MRI of PIBMA_OAm_-F_SON_-IR780 NPs with various concentrations. (b) In vivo ^1^H and ^19^F MRI images of 4T1 subcutaneous tumor-bearing BALB/c mice after intravenous (i.v.) injection with PIBMA-F_SON_-Cy7 polymer and PIBMA_OAm_-F_SON_-IR780 NPs, respectively, at different time intervals.

As expected, PIBMA_OAm_-F_SON_-IR780 NPs displayed satisfactory ^19^F MRI performance (Figures 5a and S37). After oleylamine was introduced, PIBMA_OAm_-F_SON_-IR780 NPs maintained good biocompatibility with 4T1 and MRE piC cell lines (Figure S38). Interestingly, it was found that the PIBMA-F_SON_ polymer and self-assembled PIBMA_OAm_-F_SON_-IR780 NPs exhibited different distribution behaviors after administration when using a subcutaneous tumor model. In vivo ^1^H and ^19^F MRI images of tumor-bearing mice revealed that PIBMA_OAm_-F_SON_-IR780 NPs were able to accumulate at tumor sites, whereas ^19^F MRI signals could not be substantially acquired for groups treated with PIBMA-F_SON_-Cy7 polymer (Figure 5b). However, it is important to note that the ^19^F MRI at the tumor site was still weak, which was ascribed to the poor penetration of solid tumor tissue and also because the tomoscan of MRI merely collected signals at a given layer, providing a trade-off between spatial resolution and signal intensity.

### Optical imaging of the PIBMA_OAm_-F_SON_ In Vitro and In Vivo

**Figure 6:**
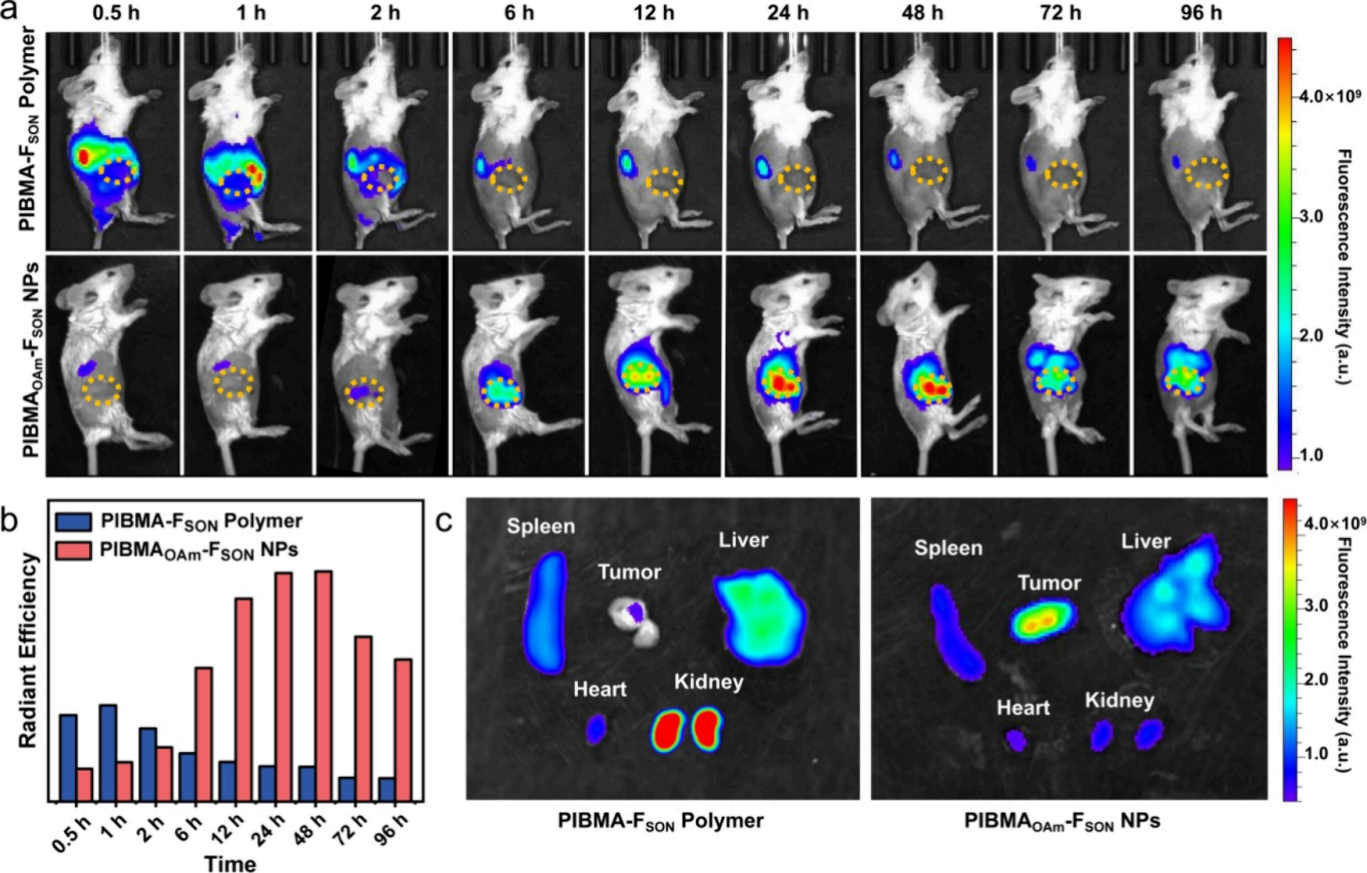
(a) In vivo NIR-fluorescence imaging of BALB/c mice with 4T1 subcutaneous tumor post intravenous (i.v.) injection with PIBMA-F_SON_-Cy7 or PIBMA_OAm_-F_SON_-IR780 NPs at different time intervals (Lateral position). (b) Relative signal intensities shown in the tumor site of mice in (a). (c) Ex vivo tissue imaging 48 h postinjection of PIBMA-F_SON_-Cy7 polymer or PIBMA_OAm_-F_SON_-IR780 NPs.

The dynamic distributions of the probes were also monitored by using optical imaging. In line with the observation from the ^19^F MRI, NIR-fluorescence imaging clearly visualized the tumor sites of mice treated with PIBMA_OAm_-F_SON_-IR780 NPs at prolonged times (Figure 6a), while for the PIBMA-F_SON_-Cy7 group, an increased accumulation at the kidney was observed at 0.5 h which then decreased gradually, suggesting that the PIBMA-F_SON_-Cy7 probe was metabolized quickly through renal clearance owing to the good aqueous solubility and the small hydrodynamic size (less than the glomerular filtration cutoff). The dynamic flow of probes into the liver, kidney, and bladder organs was supported by in vivo NIR-fluorescence imaging of the mice in the supine position (Figure S39). The quantitative comparison of fluorescence intensities at tumor sites clearly suggested the significant accumulation of PIBMA_OAm_-F_SON_-IR780 NPs (Figure 6b), which may be ascribed to the enhanced permeation retention effect (EPR) of nanosized structures with a suitable size distribution. Such enrichment was further verified by the fluorescence signals of different organs and tumor tissues 48 h postintravenous (i.v.) injection (Figure 6c). Clearly, the PIBMA-F_SON_-Cy7 probe was hardly retained at the tumor site due to its fast metabolism. Meanwhile, the strongest fluorescence intensity at the tumor site was observed for the PIBMA_OAm_-F_SON_-IR780 NPs, which confirmed the excellent enrichment of the PIBMA_OAm_-F_SON_-IR780 NPs in tumor tissue. It should be noted that while fluorescence emission was observed in the main organs, no obvious damage of the major organs (via the histological examination) was observed after treatment with PIBMA_OAm_-F_SON_-IR780 NPs (Figure S40). We also conducted a blood biochemistry assay to analyze liver and kidney function biomarkers. No significant changes in the blood level of these markers were observed at 1-week postinjection, which is indicative of no renal toxicity (Figure S41).

## Conclusions

In summary, we synthesized a polymer-based probe (PIBMA-F_SON_) with low toxicity, good hydrophilicity, and high fluorine content for ^19^F MRI with almost zero background and deep tissue penetration. The carboxylate group and the sulfoxide group endow the polymer with excellent hydrophilicity, which enables additional functionalization, including the labeling with fluorescent dyes and/or targeting peptides. The as-prepared PIBMA-F_SON_-Cy7-RGD was successfully used for in vivo ^19^F MRI and NIR fluorescence imaging of orthotopic bladder cancer. Moreover, the targeting behavior of the PIBMA-based probes could be regulated by replacing 5% of the carboxylate units on the polymer with oleylamine to form self-assembled PIBMA_OAm_-F_SON_-IR780 NPs that exhibited long *T*_2_ relaxation times (437 ms). The PIBMA_OAm_-F_SON_-IR780 NPs exhibited selective targeting toward a 4T1 subcutaneous tumor model, which may be ascribed to the EPR effect of the nanostructured probe. In this regard, we have illustrated that the rational design of ^19^F MRI probes for selective targeting is possible by regulating the local environment of the fluorinated moieties and the self-assembly behavior of polymers. Significantly, by regulation of the local hydrophilic properties and self-assembly of the ^19^F MRI probes, it was possible to construct dual-modality imaging probes with highly sensitive and selective tumor-targeting ability.

## Materials and Methods

### Materials and Reagents

NaCl, MgSO_4_, NaOH, tetrahydrofuran, dichloromethane, *N*,*N*-dimethylformamide, *n*-hexane, acetone, and diethyl ether were supplied by the Beijing Chemical Factory. Poly (isobutylene-alt-maleic anhydride) (PIBMA, 85%), hydrogen peroxide (H_2_O_2_, 30%, w/w), and oleylamine (OAm) were purchased from Sigma-Aldrich. Trifluoroiodoethane (98%) and 2-aminoethyl mercaptan hydrochloride (98%) were supplied by Beijing Innochem Science & Technology Co., Ltd. 1-Ethyl-3-(3-dimethyl aminopropyl) carbodiimide (EDC, Sigma) and N-hydroxysuccinimide (NHS, Acros) were used for bioconjugation company. Targeting peptide (Arg-Gly-Asp, RGD) was supplied by Nanjing Yuanpeptide Biotech Co.Ltd. Cyanine 7 amine was purchased from Duofluor Inc. 5-aminofluorescein was obtained from Aladdin. 11-chloro-1,1″-dipropyl-3,3,3″,3″-tetramethyl-10,12-trimethylleneindatricarbocy amine iodide (IR780) was supplied by Alfa Aesar. For cell experiments, Dulbecco’s modified Eagle’s medium (DMEM) and phosphate buffer solution (PBS) were supplied from M&C Gene Technology (Beijing) Ltd. Methylthiazolyltetrazolium (MTT) was obtained from Amresco Inc. General chemicals were of analytical grade and used as received without further purification. Deionized (DI) water was used throughout all experiments. It was obtained from a Millipore Milli-Q purification system.

### Synthesis of 2-(Trifluoroethyl) Thioethylamine

2-(Trifluoroethyl) thioethylamine (F_SN_) was synthesized following the reported procedure.^32^ In brief, 2-aminoethyl mercaptan hydrochloride (26 mmol) was dissolved into a round-bottom flask, followed by the addition of 10 mL of NaOH solution (5.2 M), and the solution was stirred for 30 min. Then, trifluoroiodoethane (38 mmol) dissolved in 30 mL of DMF was added dropwise into the flask and stirred at room temperature for 24 h. The final reaction solution was mixed with saturated sodium chloride and extracted with ethyl acetate three times, and the organic phase was collected and dried over anhydrous MgCl_2_. The volatile components were removed under a vacuum, yielding a colorless liquid as the product (2.52 g, yield 60%).

### Synthesis of PIBMA-F_SN_

Poly (isobutylene-maleic anhydride) (181 mg) was dissolved in DMF (1 mL) under magnetic stirring. Then, F_SN_ (1.0 mmol) in 1 mL of DMF was added to the mixture, and the mixture was stirred at room temperature. After 24 h, the polymer was precipitated in a mixture of dichloromethane and hexane. The precipitate was redissolved in THF and purified with hexane three times. 286 mg of the yellow solid was obtained as the product (yield 92%).

### Preparation of PIBMA-F_1_

PIBMA-F_SN_ (22 mg) was dissolved in a mixture containing 300 μL of THF and 700 μL of DCM. Then, the solution was injected into a NaOH aqueous solution (1 μM, 10 mL) under sonication. After sonicating the mixture for 6 min, the mixture was stirred at 40 °C to evaporate the organic solvent. The colloidal solution was centrifuged at 20,000 rpm for 10 min. The obtained sediment was dispersed in water for later use.

### Preparation of PIBMA-F_2_

Hydrogen peroxide was used to oxidize the thioether group on the side chain of the polymer into the sulfoxide group. The PIBMA-F_SN_ powder was dissolved in 2 mL of acetone, and 630 μL of H_2_O_2_ was added and then reacted at 40 °C for 60 h. After that, the product as a white solid was obtained through precipitation with diethyl ether.

### Synthesis of PIBMA-F_SON_

The aforementioned polymer PIBMA-F_2_ was then treated with dialysis by immersing under NaOH aqueous solution (pH = 9.0) for 48 h, which was further purified by dialysis with PBS (pH = 7.4) for 12 h. Finally, a PIBMA-F_SON_ stock solution with a concentration of 125 mg/mL was obtained *via* condensation. The as-synthesized PIBMA-F_SON_ had an absolute *M*_n_ of 10327 with a relatively low molar mass dispersity (*M*_w_/*M*_n_ = 1.2).

### Bioconjugation with RGD Peptide or Cy7-Amine

The PIBMA-F_SON_ (250 mg) was dissolved in 5 mL of water, followed by dropwise addition of MES (0.1 M) to adjust the pH value to 6.0. Then, the PIBMA-F_SON_ solution was mixed with EDC (0.025 mmol) and NHS (0.050 mmol). After the mixture was stirred for 0.5 h, 10 mg of RGD or 1 mg of Cy7-amine dissolved in PBS (pH = 7.4) was added into the PIBMA-F_SON_ solution. Finally, the resultant solution was purified using dialysis for 24 h. The obtained bioconjugated polymer solution was stored at 4 °C for later use.

### Synthesis of PIBMA_OAm_-F_SON_

Poly (isobutylene-maleic anhydride) (181 mg) was dissolved in DMF (1.0 mL) under magnetic stirring. Then, F_SN_ (1.0 mmol) and OAm (20 μL) in 1 mL of DMF were added to the mixture, and the mixture was stirred at room temperature. After reacting for 24 h, the polymer was precipitated in a mixture of DCM and hexane. The precipitate was further purified with THF and then hexane for three times. Finally, a yellow solid was obtained (140 mg). Thereafter, PIBMA_OAm_-F_SN_ (560 mg) was dissolved in 2 mL of acetone, and 800 μL of H_2_O_2_ (30%) was added and then reacted at 40 °C for 60 h. After that, the product was purified through precipitation with diethyl ether.

### Preparation of PIBMA_OAm_-F_SON_-IR780

In a typical process of ultrasonic emulsification, PIBMA_OAm_-F_SON_ (25 mg) and IR780 (0.15 μmol, 0.1 mg) were dissolved in a mixture containing 100 μL of DMSO and 300 μL of DCM. Then the solution was injected into 5 mL of NaOH aqueous solution under sonication. After sonicating for 6 min, the mixture was stirred at 40 °C to evaporate the organic solvent. The colloidal solution was centrifuged at 20,000 rpm for 10 min. The obtained sediment was dispersed in water for later use.

### MTT Assay

The cytotoxicity of the as-prepared probe was evaluated using methylthiazolyltetrazolium (MTT) assays. Briefly, about 5 × 10^4^ cells/well of 4T1 cells or MREpiC were seeded in a 96-well microtiter plate and incubated at 37 °C for 12 h to make sure that the cells adhered. Then, PIBMA-F_SON_ and PIBMA-F_SON_-RGD with different concentrations were added to the plate and cultured at 37 °C for 24 or 48 h, respectively, under 5% CO_2_ and a 95% relative humidity atmosphere. Next, 20 μL of a sterile-filtered MTT stock solution in PBS (4.0 mg/mL) was added to each well, and the plate was then incubated at 37 °C for another 4 h. The relative cell viability can be calculated by measuring absorption at 492 nm on an ELISA plate reader compared to that of the control group.

### Cell Imaging

MB49 cells were cultured at 37 °C in a 12-well cell culture plate for imaging tests. Then, the as-prepared PIBMA-F_SON_ and PIBMA-F_SON_-RGD stock solution was added into each well, respectively, with a final concentration of 400 μg/mL. The cells were incubated for different time intervals. Before imaging, the cells were washed with PBS three times.

### Hemolysis Test

The whole blood of the mouse was resuspended in a 3% sodium citrate solution. Then 200 μL of this suspension was added to each tube. Various concentrations of PIBMA-F_SON_, dispersed in PBS, were added. The tubes were incubated in a 37 °C incubator for 2 h and were centrifuged for 10 min at full speed. Lysis was determined by measuring the absorbance of the supernatant at 410 nm, reflecting the amount of hemoglobin released hemoglobin. Relative hemolysis was calculated by assuming 100% lysis of hemoglobin from the red blood cells being incubated with water.

### ^1^H and ^19^F MRI in Tubes

^1^H and ^19^F MRI phantom imaging were acquired by placing the sample solution in polypropylene PCR tubes. The ^1^H imaging parameters (FLASH) were set as follows: matrix size (MTX) was 100 × 100, and repetition time (TR) and echo time (TE) were 3000 and 6.6 ms, respectively. The field of view (FOV) was set at 40 mm × 40 mm with a slice thickness of 1 mm. The ^19^F imaging parameters (*T*_1_-REAR) were set as follows: matrix size (MTX) was 100 × 100, and repetition time (TR) and echo time (TE) were 5000 and 9.28 ms, respectively. The field of view (FOV) was set at 40 × 40 mm.

### Bladder Cancer Model of C57BL/6 Mice

All mice received care following the guidelines of the Care and Use of Laboratory Animals, and their use followed the terms of the Institutional Animal Care regulations and Use Committee of China-Japan Friendship Hospital and Beijing University of Chemical Technology. C57BL/6 mice under anesthetization were injected with MB49 cells (2 × 10^6^) into their bladder wall via a 0.7 × 19 mm i.v. Cannula syringe. One month after cell inoculation, the bladder tumors could be imaged by ^1^H MRI, confirming the successful establishment of a bladder cancer model. After 6 weeks, tumor-bearing mice were intravenously injected with 200 μL (125 mg/mL) of PIBMA-F_SON_ or PIBMA-F_SON_-RGD solution. The in vivo MRI was carried out after the mouse was anesthetized with isoflurane.

### Subcutaneous Tumor Model of Balb/c Mice

First, the 4T1 cells were centrifuged at 1000 rpm for 5 min and resuspended in physiological saline to a final concentration of 1 × 10^7^ cells/mL. Then, 4T1 cells (1 × 10^6^ cells) were subcutaneously inoculated on the right leg of 4-week-old female mice (Balb/c). As the volume of tumor grew up to 300 mm^3^, tumor-bearing mice were intravenously injected with 200 μL of PIBMA-F_SON_-Cy7 polymer or PIBMA_OAm_-F_SON_-IR780 NPs solution. In vivo MRI measurements were performed with mice anesthetized by isoflurane.

### In Vivo Experimental Procedure for MRI and Fluorescence Imaging

The *T*_1_-RARE sequence was used for ^19^F MRI measurements, and the related parameters were set as follows: matrix size (MTX) was 100 × 100, and repetition time (TR) and echo time (TE) were 1200 and 4.64 ms, respectively. The field of view (FOV) was set at 40 × 40 mm with a slice thickness of 5 mm. The total ^19^F MRI experiment time was 19 min. The *T*_1_ RARE sequence was used for ^1^H MRI, and the related parameters were set as follows: matrix size (MTX) was 100 × 100, and repetition time (TR) and echo time (TE) were 4529 and 40 ms, respectively. The field of view (FOV) was set at 40 × 40 mm with a slice thickness of 1 mm. The total ^1^H MRI experiment time was about 3 min 1 s. Those mice were imaged by the small animal live imaging system from the PE company (IVIS Spectrum) for fluorescence imaging.

### Serological and Histological Analysis

The blood samples and major organs of mice injected with 100 μL of PIBMA_OAm_-F_SON_-IR780 solution and healthy mice as control, respectively, were collected 1 week after administration for blood biochemical analysis and histological examination. All experiments were carried out at least in triplicate, and results were expressed as means ± SD.

## Supporting Information Available

NMR spectra of F_SN_ molecule and PIBMA-F_SN_ polymer; TEM and DLS size distribution of PIBMA-F_1_; conversion of PIBMA-F_SON_; ^1^H NMR and FTIR spectra of PIBMA-F_SON_; the photo of PIBMA-F_SN_, PIBMA-F_1_, PIBMA-F_2_, and PIBMA-F_SON_; the zeta potential of PIBMA-F_1_ NPs and PIBMA-F_SON_; stability investigation of PIBMA-F_SON_; biocompatibility measurement; cell targeting studies and NIR-fluorescence images of PIBMA-F_SON_-Cy7 and PIBMA-F_SON_-Cy7-RGD; serological and histological analysis of PIBMA-F_SON_; chemical structure and morphology investigation of PIBMA_OAm_-F_SON_; NIR-fluorescence images and ^19^F NMR of PIBMA_OAm_-F_SON_-IR780 NPs; zeta potential and stability tests of PIBMA_OAm_-F_SON_-IR780 NPs; in vitro ^19^F MRI, cell viability, in vivo fluorescence imaging, and biosafety of PIBMA_OAm_-F_SON_-IR780 NPs (PDF)

## Abbreviations

(MRI): magnetic resonance imaging
(PIBMA): poly (isobutylene-maleic anhydride)
(PFC): perfluorocarbon
(PFCE): perfluoropolyether
(FSN): 2-(trifluoroethyl) thioethylamine
(Cy7): heptamethine cyanine dye derivatives
(Arg-Gly-Asp, RGD): a targeting peptide
(NPs): nanoparticles
(Peakw): half peak width
(SNR): signal-to-noise ratio
(TEM): transmission electron microscopy
(DLS): dynamic light scattering
(FTIR): Fourier transform infrared
