# Supplementary material for: Superhydrophilic Fluorinated Polymer Probe for Zero-Background 19F MRI with Adaptable Targeting Ability
Source: ACS Appl Mater Interfaces. 2024 Nov 15;16(47):65319–27. doi: 10.1021/acsami.4c14715 (PMC11620481; doi:10.1021/acsami.4c14715)
Supplement: Supplementary file 1 — am4c14715_si_001.pdf [file am4c14715_si_001.pdf]

Supporting Information

Superhydrophilic Fluorinated Polymer Probe for Zero-Background ^{19}F MRI with Adaptable Targeting Ability

Chang Guo[‡a], Xiaoyao Xiong[‡a], Xinxing Zhao[‡a], Yumin Li[a], Sijia Li[a], Suying Xu[a], Tony D. James*[b,c] and Leyu Wang*[a]*

[a] State Key Laboratory of Chemical Resource Engineering, College of Chemistry, Beijing University of Chemical Technology, Beijing 100029, China.

[b] Department of Chemistry, University of Bath, BA2 7AY, Bath, United Kingdom.

[c] School of Chemistry and Chemical Engineering, Henan Normal University, Xinxiang 453007, China.

* syxu@mail.buct.edu.cn; t.d.james@bath.ac.uk; lywang@mail.buct.edu.cn.

[‡]These authors contributed equally to this work.

Table of Contents

1. Instrumentation Section 3

2. Supporting Figures..4

3. Supporting References 24

1. Instrumentation Section

TEM images were obtained by using a JEOL JEM-1200EX (100 kV). Dynamic light scattering (DLS) particle size analysis was carried out using a Zetasizer Nano-ZS90 zeta and size analyzer from Malvern. Fourier transform infrared (FTIR) spectra were acquired on a Nicolet 6700 FTIR spectrophotometer (Thermo Fisher). Gel permeation chromatography (GPC) analysis was taken on Waters 1525 high-performance liquid chromatography equipped with a 2414 refractive index detector. The chemical structure of the polymer was measured by ^{1}H magnetic resonance spectra recorded on a Bruker Avance-III 400 MHz NMR spectrometer at 298 K. The ^{19}F magnetic resonance spectra were recorded on a Bruker Avance-III 400 MHz NMR spectrometer by utilizing a "single-pulse" sequence (a Bruker "zg" sequence) without decoupling of ^{1}H. A coaxially D_2O-filled capillary tube was used for locking the field. The ^{19}F NMR relaxation time was measured on Bruker Avance-III 400 MHz NMR spectrometer. The standard inversion-recovery (t1ir) pulse sequence and Carr-Purcell-Meiboom-Gill (CPMG) pulse sequence were used to measure ^{19}F spin-lattice relaxation (T_1) and spin−spin relaxation (T_2), respectively, during which the sample temperature was maintained at 298 K. The ^{19}F NMR relaxation time measurements of PIBMA-F_{SON} with different concentrations were carried out on a 7.0 T Bruker Bio-Spec70/20USR MRI system. T_1 map-RARE and T_2 map-MSME pulse sequences were used to measure ^{19}F spin-lattice relaxation (T_1) and spin−spin relaxation (T_2), respectively. Cell fluorescence imaging was performed on an ECLIPSE Ti2 Microscope (Nikon). Fluorescence imaging was performed on a small animal live imaging system from PE company (IVIS Spectrum).

2. Supporting Figures

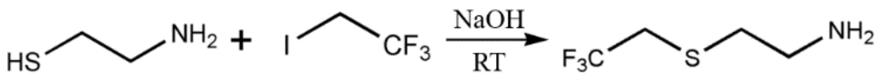

Figure S1. Synthetic route of 2-(trifluoroethyl) thioethylamine (FSN).

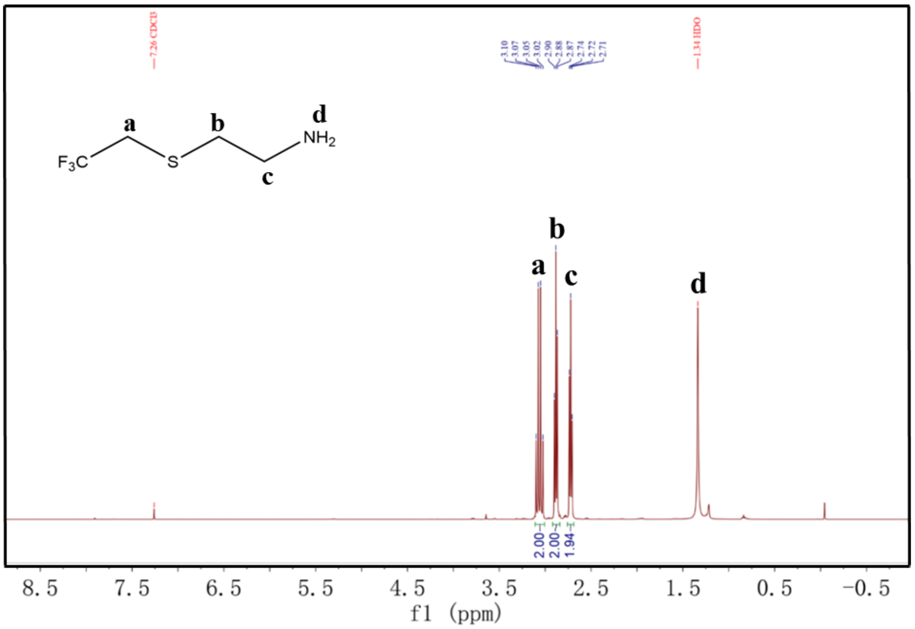

Figure S2. ^{1}H NMR spectrum of compound FSN (400 MHz, Chloroform-*d*) δ (ppm): 3.06 (q, *J* = 9.9 Hz, 2H), 2.88 (t, *J* = 6.3 Hz, 2H), 2.72 (t, *J* = 6.3 Hz, 2H).

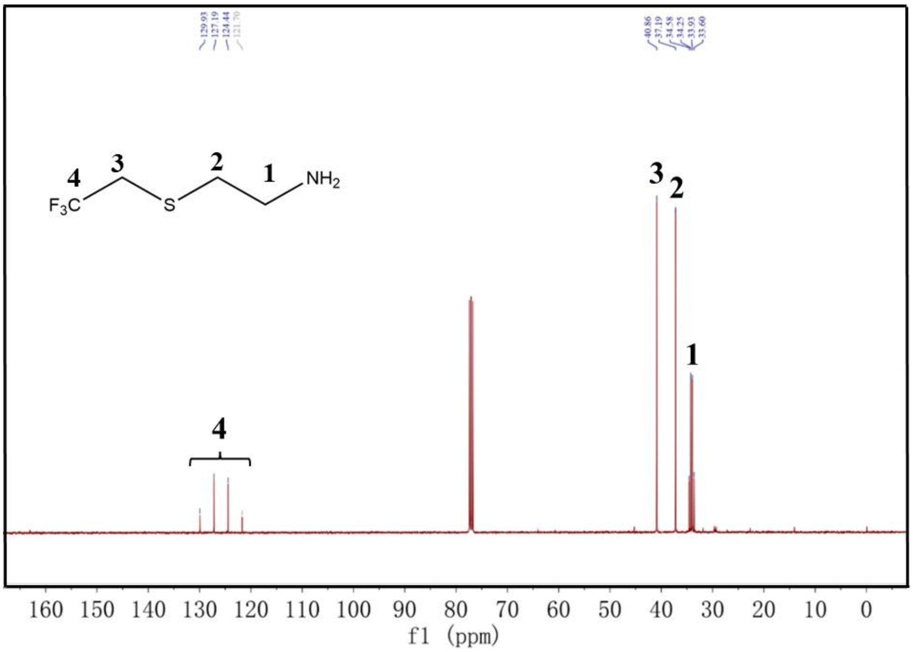

Figure S3. ^{13}C NMR spectrum of compound F_{SN} ^{13}C NMR (101 MHz, Chloroform-*d*) δ (ppm): 125.81 (q, *J* = 276.2 Hz), 40.86, 37.19, 34.09 (q, *J* = 32.7 Hz).

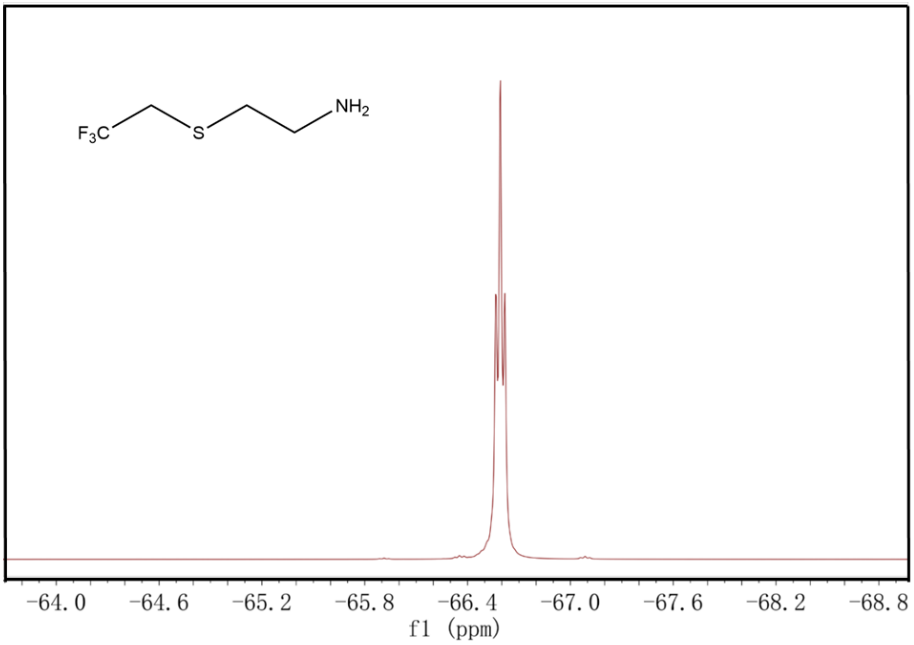

Figure S4. ^{19}F NMR spectrum of compound F_{SN} ^{19}F NMR (376 MHz, Chloroform-*d*) δ (ppm): - 66.58 ppm.

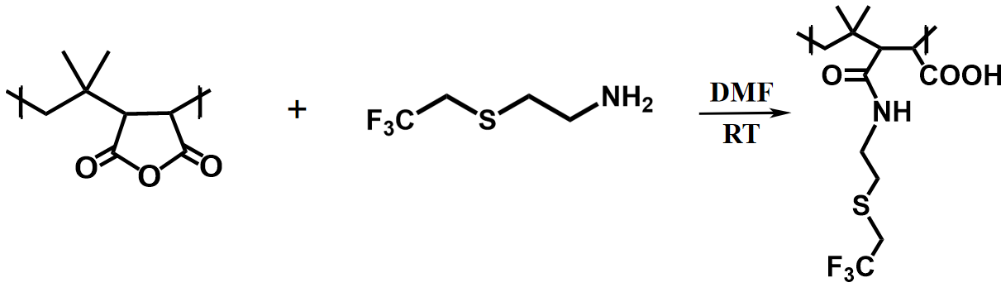

Figure S5. Synthetic route of polymer PIBMA-F_{SN}. RT: room temperature.

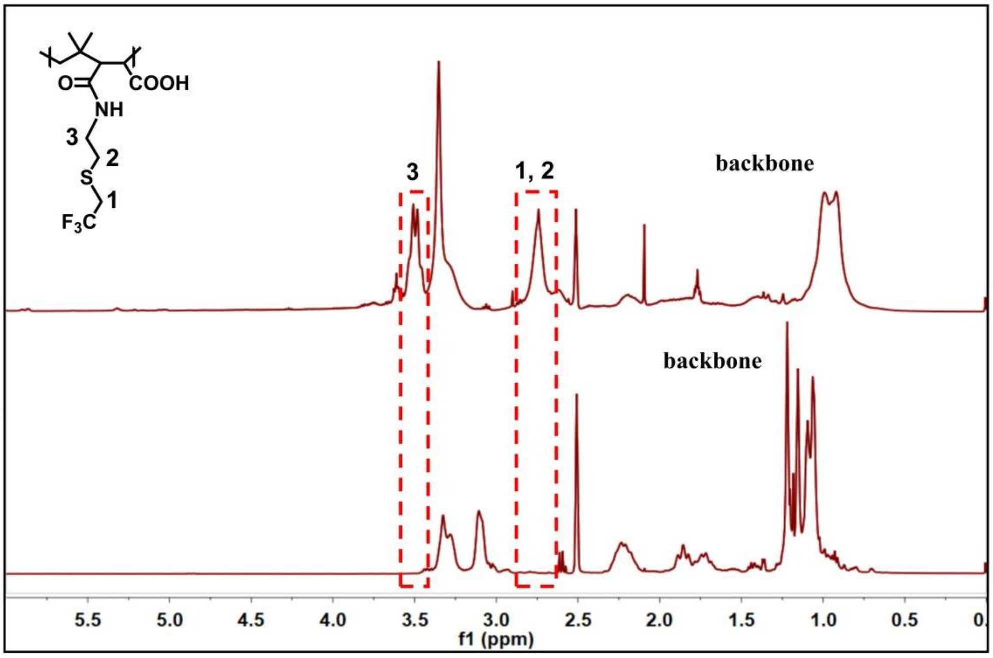

Figure S6. ^{1}H NMR spectra of PIBMA-F_{SN} (top) and PIBMA (bottom) (DMSO-d_6).

The ring-opening reaction of PIBMA with the amino group of 2-(trifluoroethyl) thioethylamine (F_{SN}) was conducted to prepare fluoropolymers containing fluorine-containing fragments and a large number of carboxyl groups, which rendered the polymer with a strong ^{19}F MRI signal and excellent water-solubility. The characterization results are shown in Figure S6. The ^{1}H NMR spectra show that the ring-opened polymer has a new proton peak of an amide at δ 3.5 ppm and a new proton peak of a methylene at δ 2.7-2.9 ppm, in comparison with the raw PIBMA (red box), implying the successful preparation of the PIBMA-F_{SN} polymer. The number-averaged (Mn) molecular weight was found to be 10,327, and the weight-averaged molecular weight (Mw) was 12,683. The polydispersity index (PDI-Mw/Mn) was calculated to be 1.23.

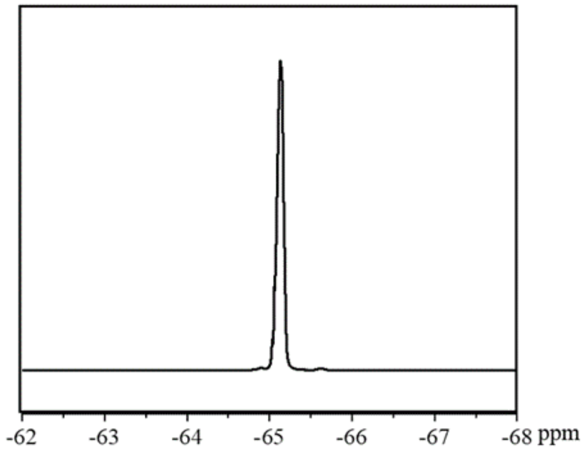

Figure S7. ^{19}F NMR spectrum of PIBMA-F_{SN} (DMSO-d_6).

The ^{19}F MRI performance of the polymer PIBMA-F_{SN} was evaluated by ^{19}F NMR. As shown in Figure S7, the as-prepared polymer PIBMA-F_{SN} has a narrow half-peak width (33.56 Hz) in DMSO, suggesting this polymer has great potential as a novel ^{19}F MRI probe.

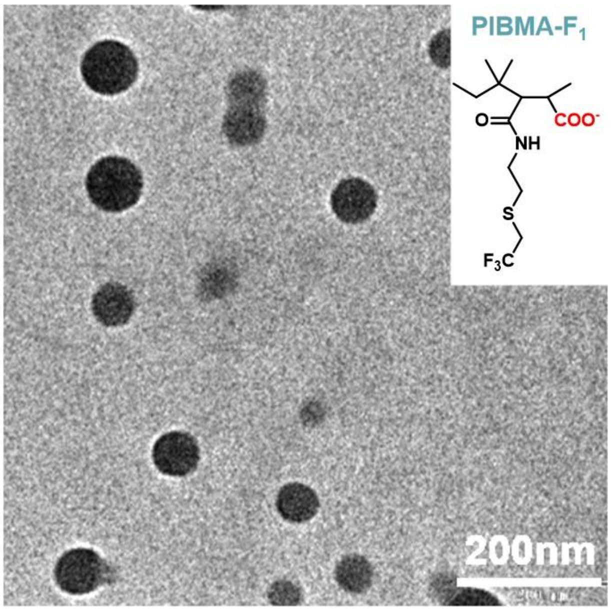

Figure S8. TEM image of PIBMA-F_1 nanoparticles. Inset is the chemical structure of PIBMA-F_1. Due to the enriched carboxylic acids, the amphiphilicity of PIBMA-F_1 makes it possible to self-assemble into nanoparticles.

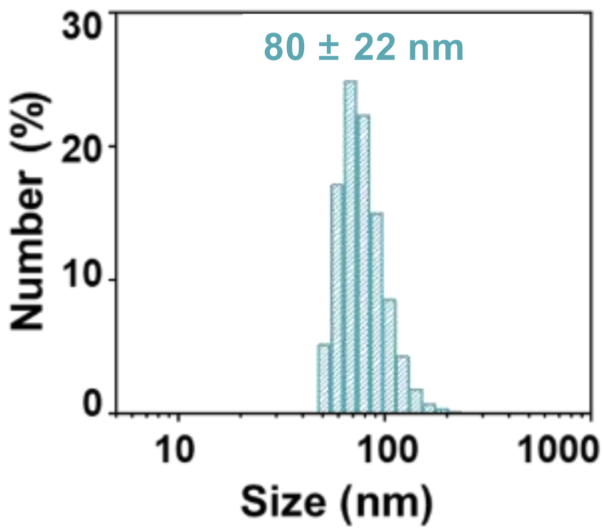

Figure S9. DLS size distribution of PIBMA-F_1 nanoparticles.

The polymer PIBMA-F_1 was self-assembled into nanoparticles. The TEM image (Figure S8) and dynamic light scattering (DLS) results (Figure S9) indicated that the PIBMA-F_1 nanoparticles are spherical with an average DLS diameter of 80 ± 22 nm.

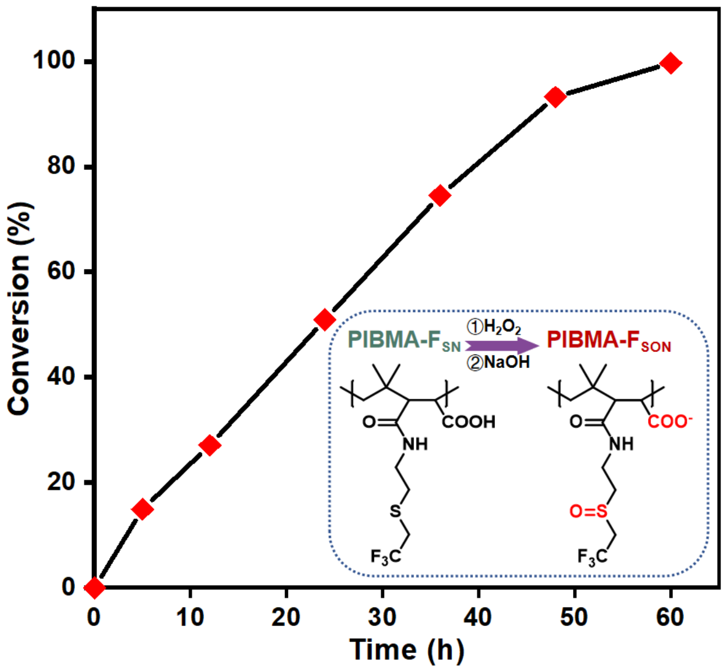

Figure S10. Conversion ratio of PIBMA-F_{SN} into PIBMA-F_{SON} as a function of oxidation time. Inset is the conversion scheme of PIBMA-F_{SN} into PIBMA-F_{SON} by H_2O_2 oxidation, followed by dispersion in NaOH.

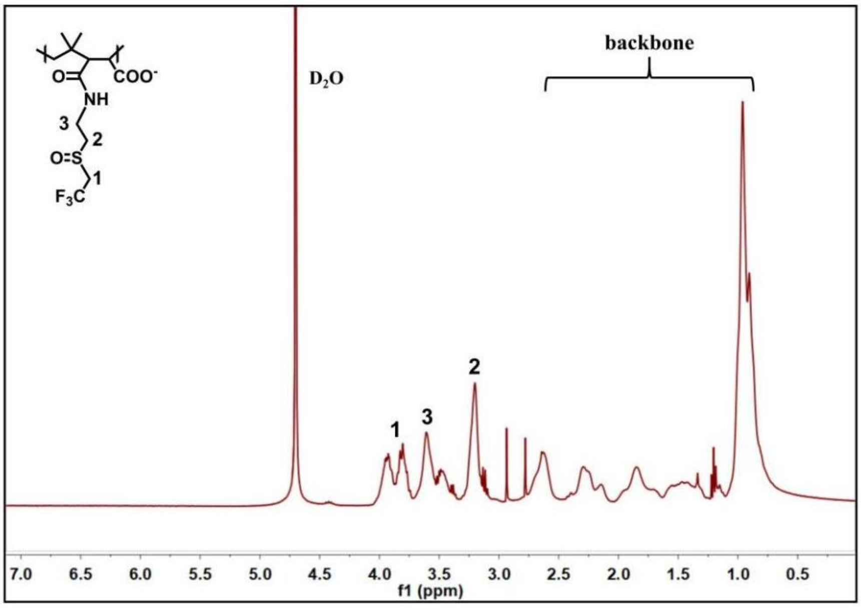

Figure S11. ^{1}H NMR spectrum of PIBMA-F$_{SON}$ (D_2O).

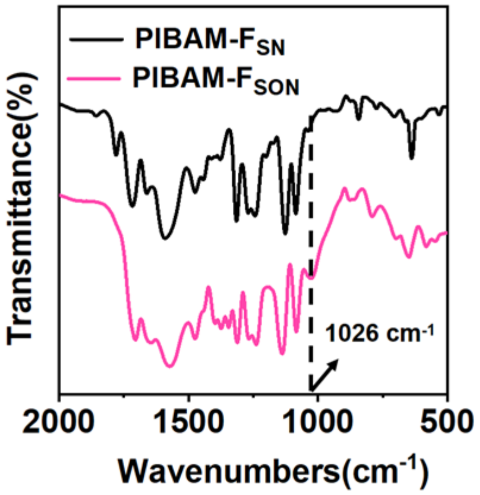

Figure S12. FTIR spectra of PIBMA-F$_{SN}$ and PIBMA-F$_{SON}$, respectively.

The PIBMA-F$_{SN}$ polymer was oxidized with hydrogen peroxide, and the oxidation process was monitored by ^{19}F NMR on basis of the chemical shift of -CF_3. The peak position of -CF_3 connected to the thioether group before oxidation was around δ -66.0 ppm, which would shift to -60.6 ppm after oxidation. The conversion ratio from thioether to sulfoxide can be evaluated by calculating the integral areas of these two peaks. As can be seen from Figure S10, the oxidation was finished at around 60 h with a conversion rate of 99%. As shown in Figure S6 and Figure S11, the ^{1}H NMR spectrum of PIBMA-F$_{SON}$ showed a series of peaks at 3.8 - 4.1 ppm, belonging to the methylene group attached to the sulfoxide group. These results indicate that the thioether group of PIBMA-F$_{SN}$ was successfully oxidized to the sulfoxide by H_2O_2.

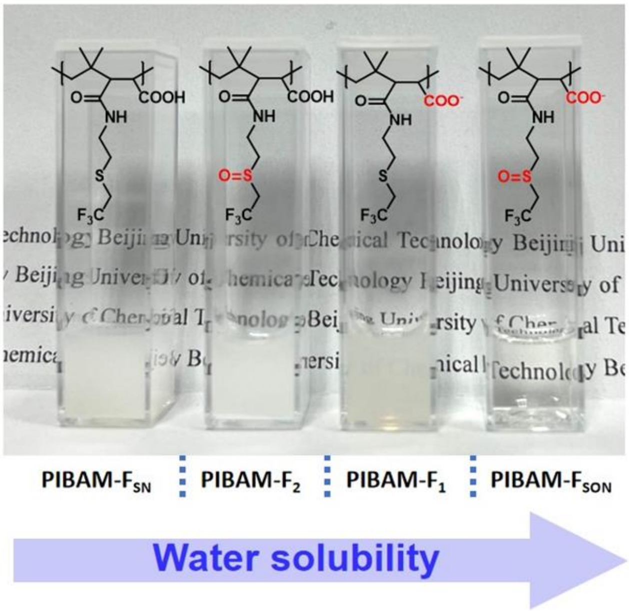

Figure S13. Photos of PIBMA-F_{SN}, PIBMA-F_2, PIBMA-F_1 and PIBMA-F_{SON} in aqueous solution, respectively.

From these photos, it is clear that, with the increase of hydrophilicity, the aqueous solution of polymer becomes transparent, implying excellent water-solubility.

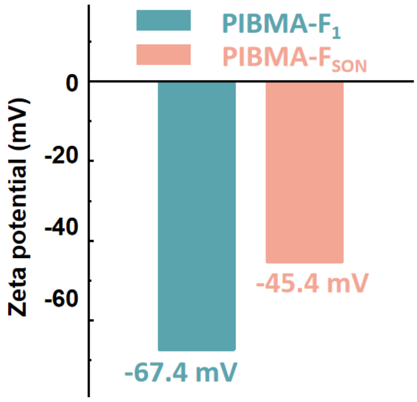

Figure S14. The zeta potential of PIBMA-F_1 NPs and PIBMA-F_{SON}.

Both PIBMA-F_1 NPs and PIBMA-F_{SON} could be dispersed in an aqueous solution. In Figure S14, the zeta potentials of PIBMA-F_1 NPs and PIBMA-F_{SON} were -67.4 and -45.4 mV, respectively. Generally, particles with zeta potentials more negative than -30 mV or more positive than +30 mV are considered stable systems due to electrostatic repulsion.[1]

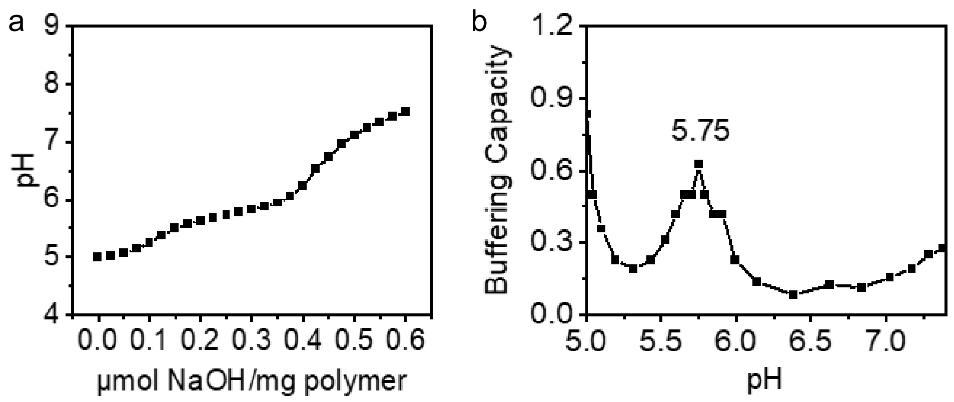

Figure S15. (a) Titration curve of PIBMA-F_{SON}. (b) The buffering capacity of PIBMA-FSON in the pH range (5.0-7.5), buffering capacity at a pH value was calculated from the inverse slope of the titration curve as Δ(NaOH)/ΔpH.

We measured the p*K*a value of PIBMA-FSON by pH titration according to previous literature.[2-4] In brief, the pH of the PIBMA-FSON solution (20 mg/mL, 2.0 mL) was adjusted to 5.0 by using 1.0 M HCl. It is important to note that the probe remained in solution without any precipitation at this pH. Subsequently, 0.10 M NaOH was gradually added and the pH of the solution was measured using a FE28 pH Meter (Mettler Toledo). **Figure S15** illustrates a peak buffering capacity of PIBMA-FSON at pH 5.75, which represents the effective p*K*a of PIBMA-FSON.

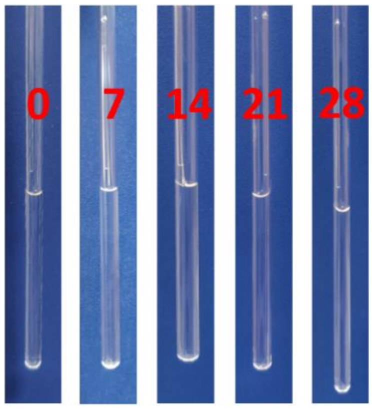

Figure S16. Photos of PIBMA-FSON solution (in PBS, pH 7.4) for different days.

As shown in these photos above, the aqueous solution of PIBMA-FSON demonstrates good water-solubility with no precipitation.

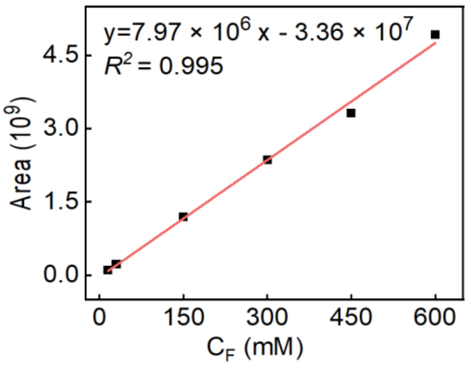

Figure S17. The plot of ^{19}F NMR integral area *versus* ^{19}F concentration (C_F/mM).

The ^{19}F NMR peak areas of a series of PIBMA-F_{SON} were measured, which could be further used to calculate the content of fluorine atoms (Figure S17). The ^{19}F concentration (C_F) of the as-prepared PIBMA-F_{SON} (20.0 mg/mL, 500 μL) was determined to be 182 mM. Thus, the mass of fluorine atom was 1.73 mg, that is, the molecular weight fraction of fluorine atom was 17%.

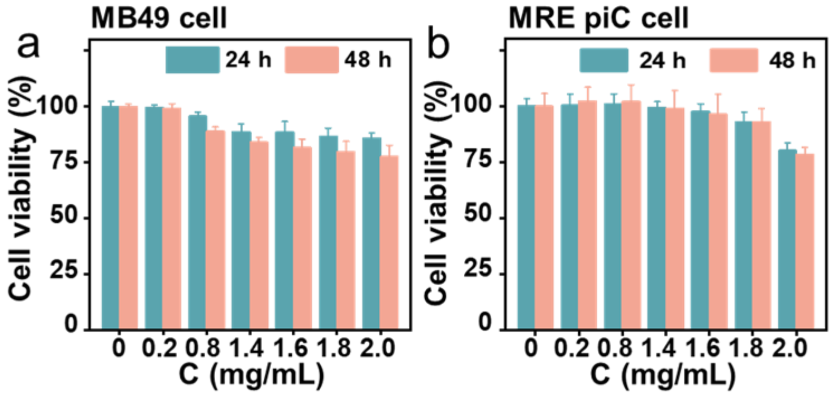

Figure S18. Cell viability of PIBMA-F_{SON} on MB49 (a) and MRE piC (b) cell lines at different concentrations after incubation for 24 h and 48 h, respectively.

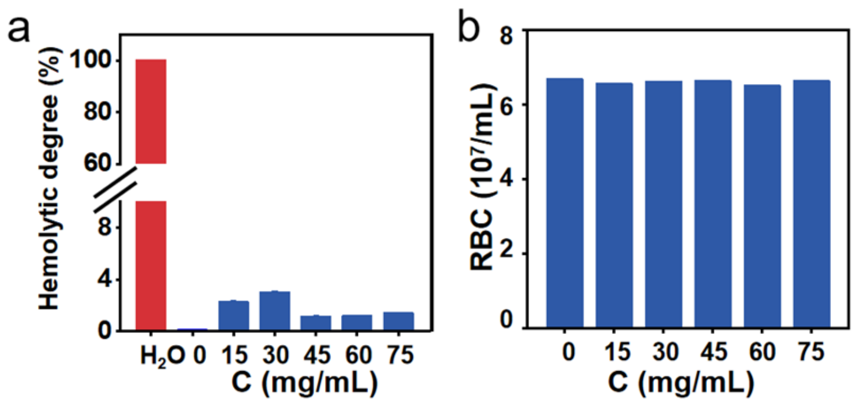

Figure S19. Hemolytic degree (a) and cell viability of the red blood cells (b) incubated with different concentrations of PIBMA-F_{SON}.

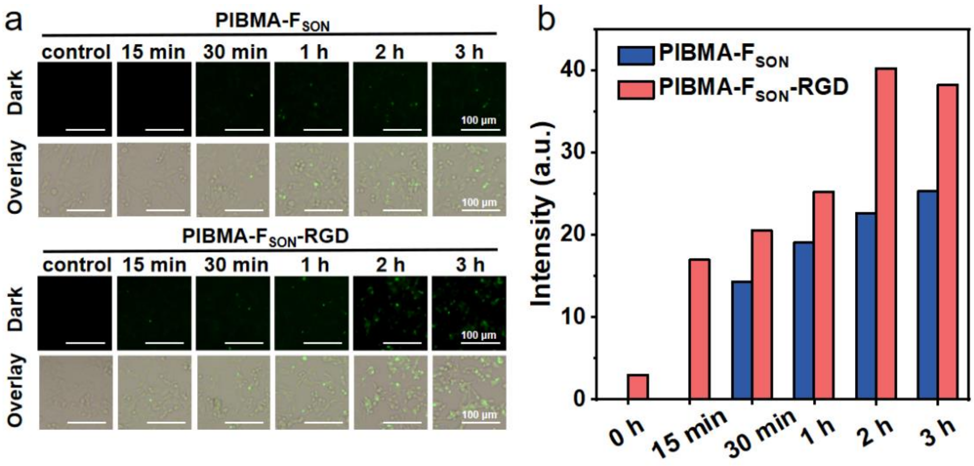

Figure S20. Fluorescence images (a) and fluorescence intensity histogram (b) of MB49 cells incubated with PIBMA-F_{SON} and PIBMA-F_{SON}-RGD labeled with 5-amino fluorescein. (Ex: 465-495 nm, Em: 512-558 nm).

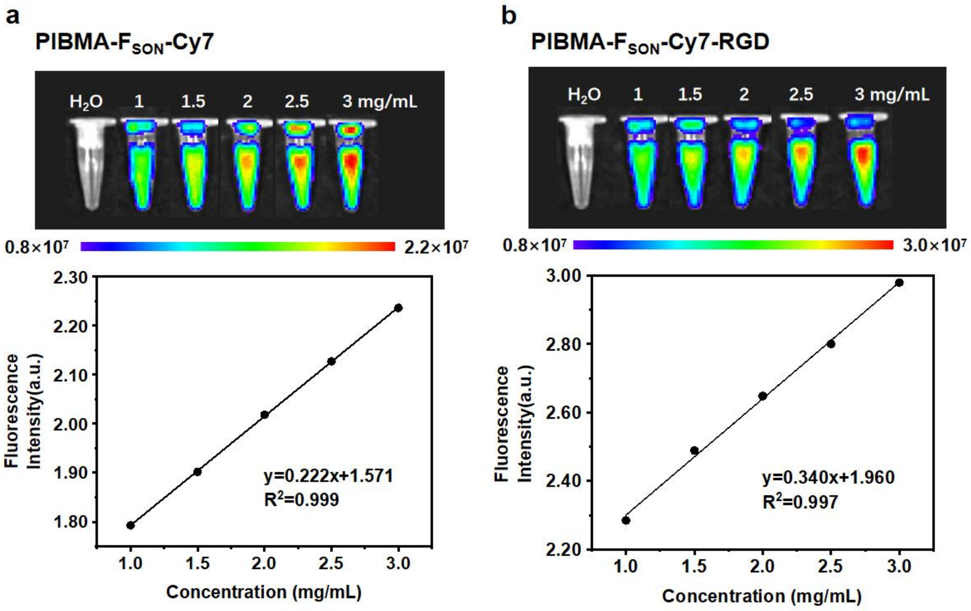

Figure S21. NIR fluorescence imaging and corresponding imaging contrast intensities of polymer PIBMA-F_{SON}-Cy7 (a) and PIBMA-F_{SON}-Cy7-RGD (b) under various concentrations (1.0 to 3.0 mg/mL). NIR-fluorescence images at 800 nm, obtained by an imaging system with an excitation wavelength at 710 nm.

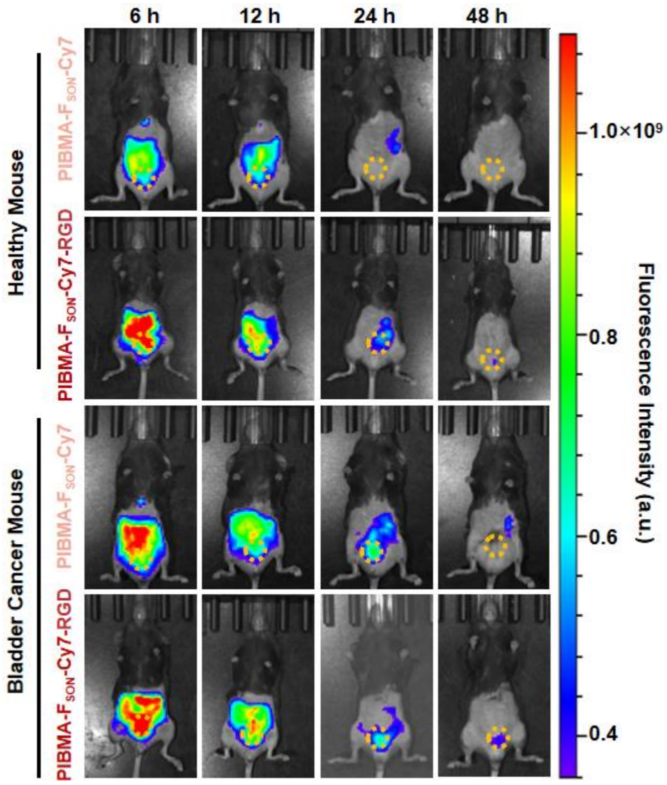

Figure S22. *In vivo* NIR-fluorescence imaging of healthy mouse and orthotopic bladder cancer model in C57BL/6 mouse post intravenous (*i.v.*) injection with PIBMA-F_{SON}-Cy7-RGD and PIBMA-F_{SON}-Cy7 at different time intervals, respectively. The yellow circle in each image indicates the tumor site.

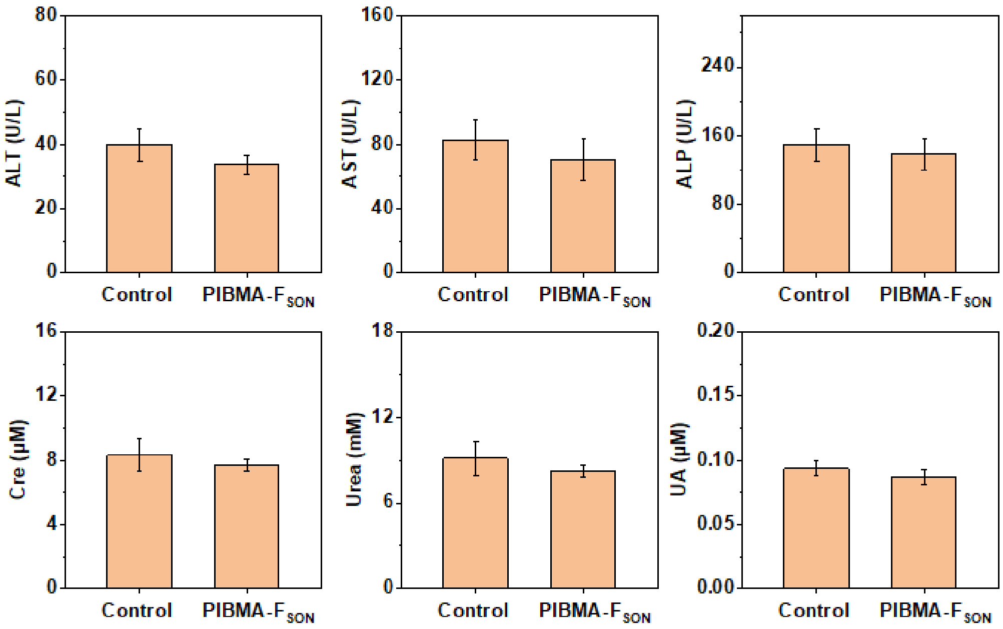

Figure S23. Serum biochemistry assay to evaluate the ALT, AST, ALP, Cre, Urea and UA levels in mice receiving i.v. injection of PIBMA-F_{SON}. Data are presented as mean ± SD (n = 3). Blood was obtained by eyeball blood sampling.

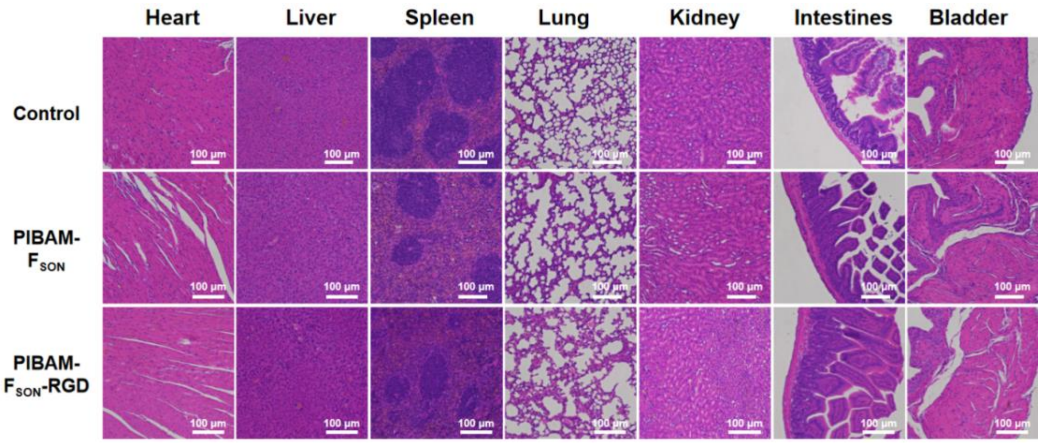

Figure S24. H&E-stained slices of main organs including heart, liver, bladder, spleen, lung, and kidney from different groups of mice.

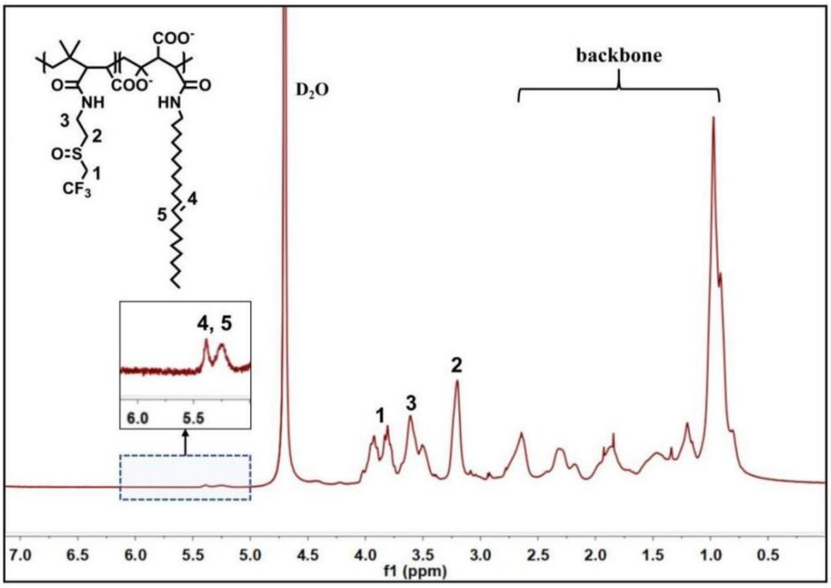

Figure S25. ^{1}H NMR spectra of $PIBMA_{OAm}$- F_{SON} (D_2O).

The ^{1}H NMR spectrum of $PIBMA_{OAm}$-F_{SON} showed a series of peaks at 3.5 - 4.0 ppm belonging to the peak of the methylene group attached to the sulfoxide group, and the proton peak at δ 5.35 ppm belonging to the double bond proton of OAm (Figure S25). The successful transformation to sulfoxide groups was demonstrated using FTIR spectroscopy (Figure S26), where the sulfoxide S=O stretching vibration at 1022 cm^{-1} was observed for $PIBMA_{OAm}$-F_{SON}.

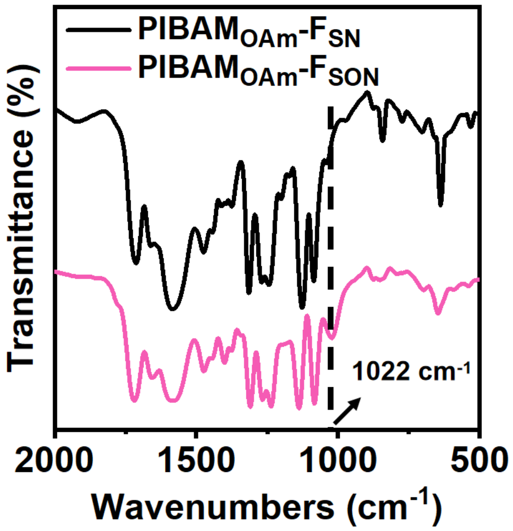

Figure S26. FTIR spectra of PIBMA$_{OAm}$-F$_{SN}$ and PIBMA$_{OAm}$-F$_{SON}$.

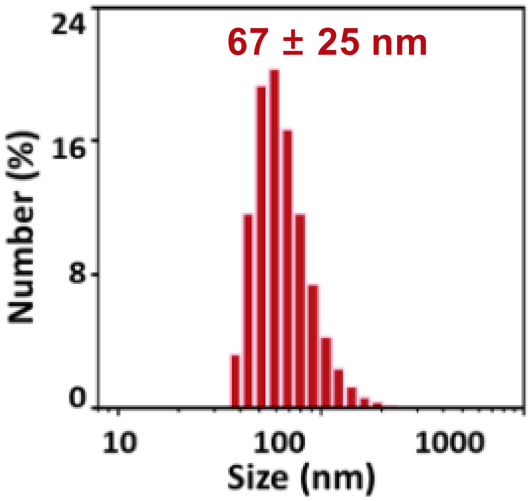

Figure S27. DLS size distribution of PIBMA$_{OAm}$-F$_{SON}$-IR780 NPs.

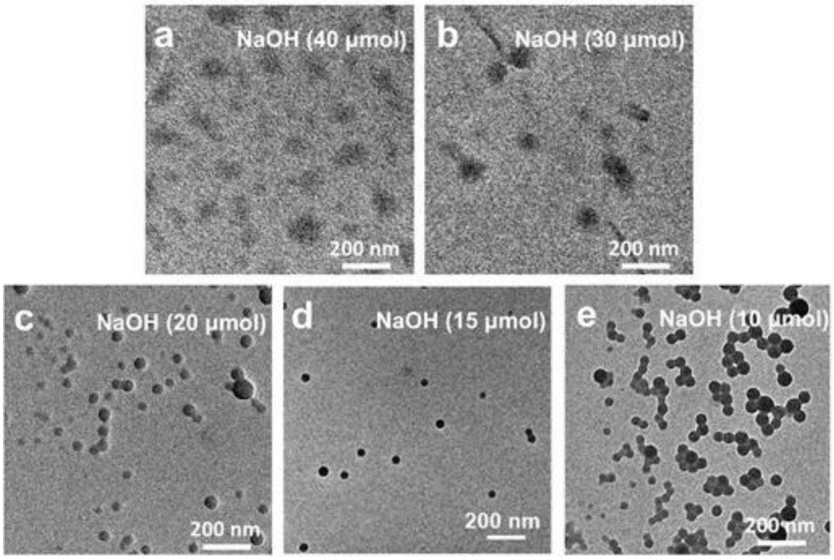

Figure S28. TEM images of PIBMA$_{OAm}$-F$_{SON}$-IR780 NPs prepared with different dosages of NaOH, (a) 40 μmol, (b) 30 μmol, (c) 20 μmol, (d) 15 μmol, and (e) 10 μmol, respectively.

PIBMA$_{OAm}$-F$_{SON}$ (25 mg) and IR780 were dissolved in a mixture containing 100 μL of DMSO and 300 μL of DCM. Then the solution was injected into 5 mL of NaOH aqueous solution under sonication. By varying NaOH dosage in the range of 10 – 40 μmol, the product changed from amorphous flocculates into regular spherical nanoparticles (Figure S28). Finally, the dosage of NaOH was optimized to 15 μmol. Under optimal condition, the loading of IR780 was explored within a range of 0.05–0.20 mg. As the loading increased, the NIR fluorescence intensity (Figure S29) and nanoparticle size (Figure S30) of the product increased gradually. Considering that nanoparticles with size less than 100 nm are optimal for *in vivo* imaging as too large particle size may lead to severe accumulation in the liver or spleen, the dosage of IR780 was optimized to 0.10 mg.

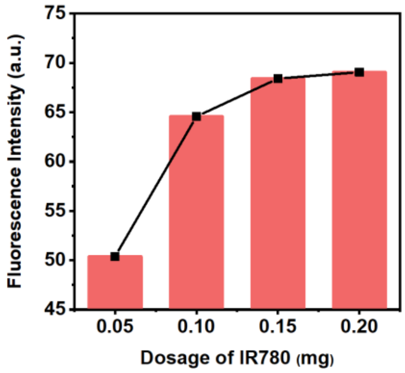

Figure S29. NIR-fluorescence intensity of PIBMA$_{OAm}$-F$_{SON}$-IR780 NPs prepared with different dosages of IR780.

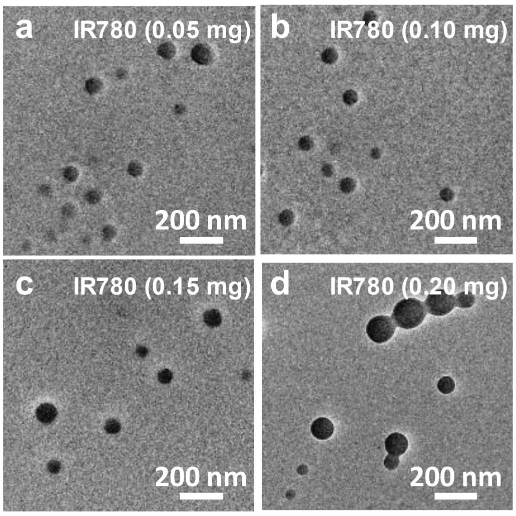

Figure S30. TEM images of PIBMA$_{OAm}$-F$_{SON}$-IR780 NPs prepared with different dosages of IR780, (a) 0.05 mg, (b) 0.10 mg, (c) 0.15 mg, and (d) 0.20 mg, respectively.

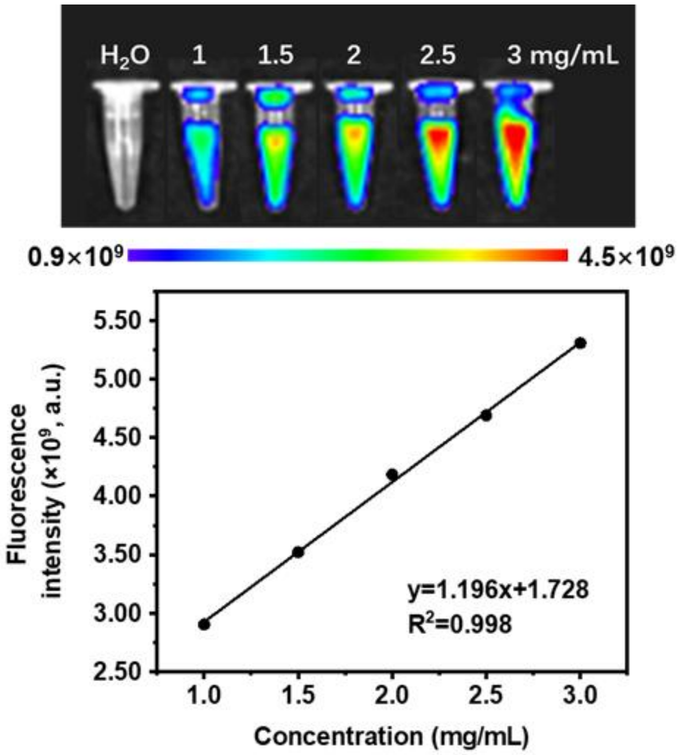

Figure S31. NIR-fluorescence images of PIBMA$_{OAm}$-F$_{SON}$-IR780 NPs at 820 nm, obtained by imaging system with excitation wavelength at 745 nm (top) and corresponding imaging contrast intensities of PIBMA$_{OAm}$-F$_{SON}$-IR780 NPs (bottom).

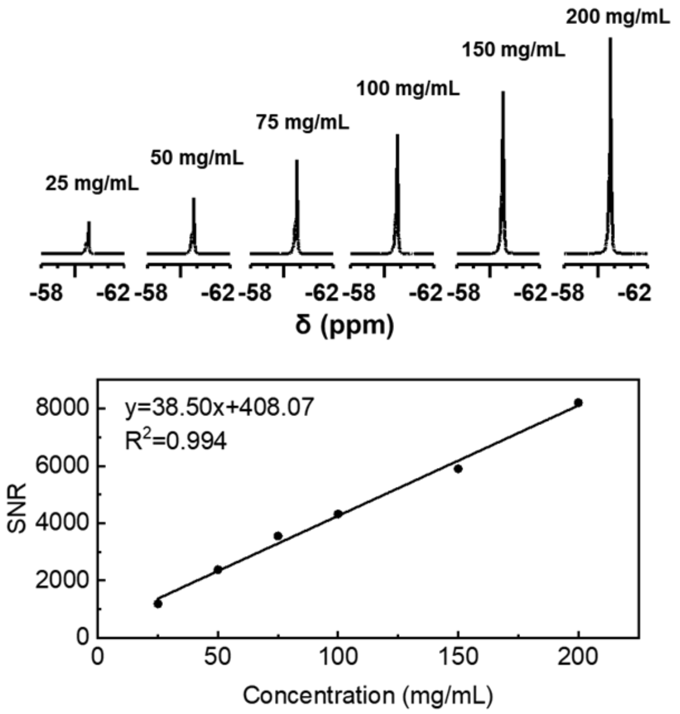

Figure S32. ^{19}F NMR spectra of PIBMA$_{OAm}$-F$_{SON}$-IR780 NPs under different concentrations. The plot of the ^{19}F NMR signal-to-noise ratio (SNR) *versus* concentration of PIBMA$_{OAm}$-F$_{SON}$-IR780 NPs.

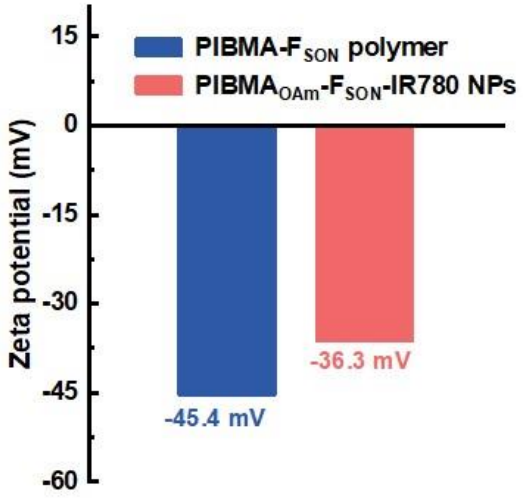

Figure S33. Zeta potentials of PIBMA-F$_{SON}$ and PIBMA$_{OAm}$-F$_{SON}$-IR780 NPs.

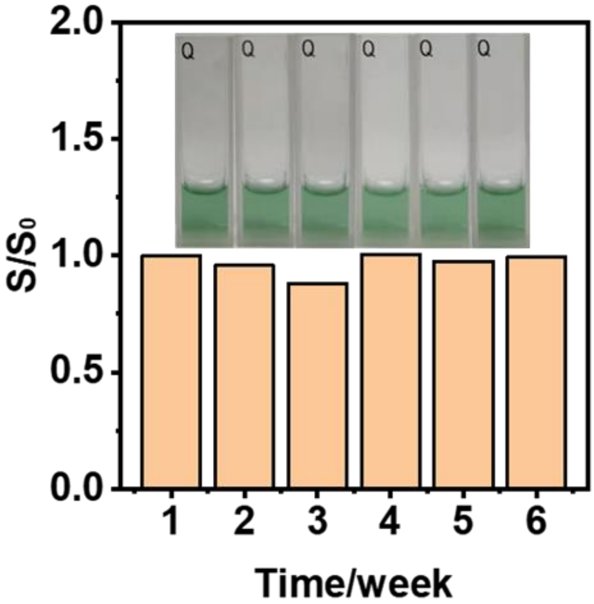

Figure S34. The evolution of ^{19}F NMR signal intensity along with time for investigating the stability of PIBMA$_{OAm}$-F$_{SON}$-IR780 NPs dispersed in PBS (pH 7.4). Insets are corresponding photographs of PIBMA$_{OAm}$-F$_{SON}$-IR780 NPs dispersed in PBS (pH 7.4) and no precipitation was observed.

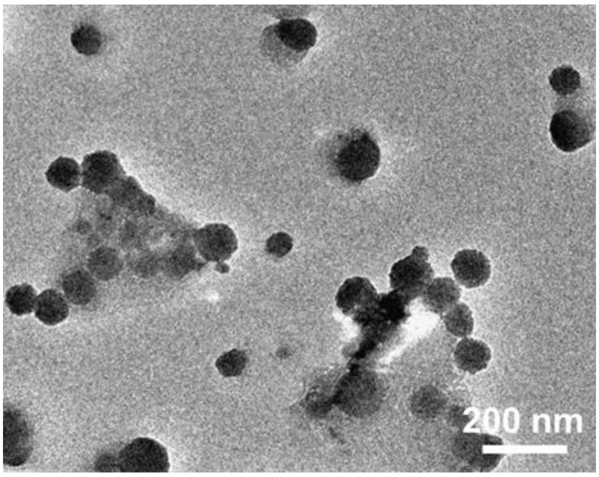

Figure S35. TEM image of redissolved PIBMA$_{OAm}$-F$_{SON}$-IR780 NPs powder.

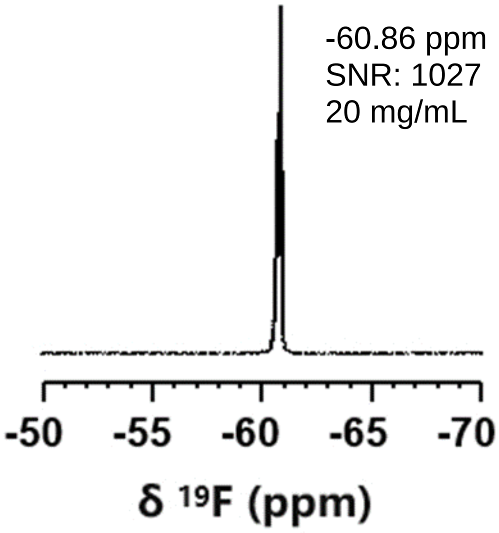

Figure S36. ^{19}F NMR of redisolved PIBMA$_{OAm}$-F$_{SON}$-IR780 NPs powder. The ^{19}F NMR signal has no observable difference in comparison with the freshly prepared PIBMA$_{OAm}$-F$_{SON}$-IR780 NPs.

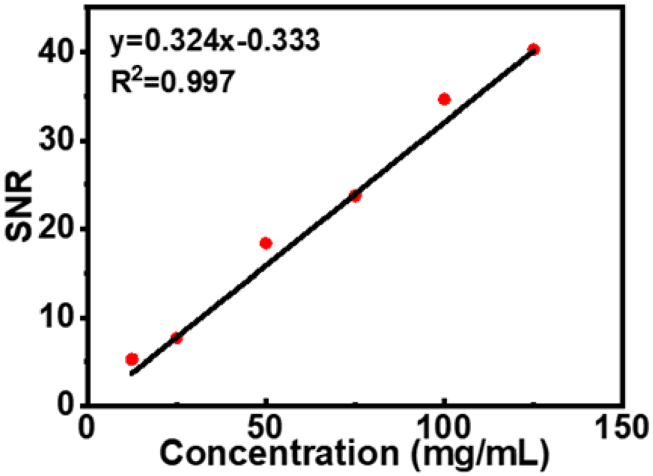

Figure S37. The plot of ^{19}F MRI signal-to-noise ratio (SNR) *versus* the concentration of PIBMA$_{OAm}$-F$_{SON}$-IR780 NPs.

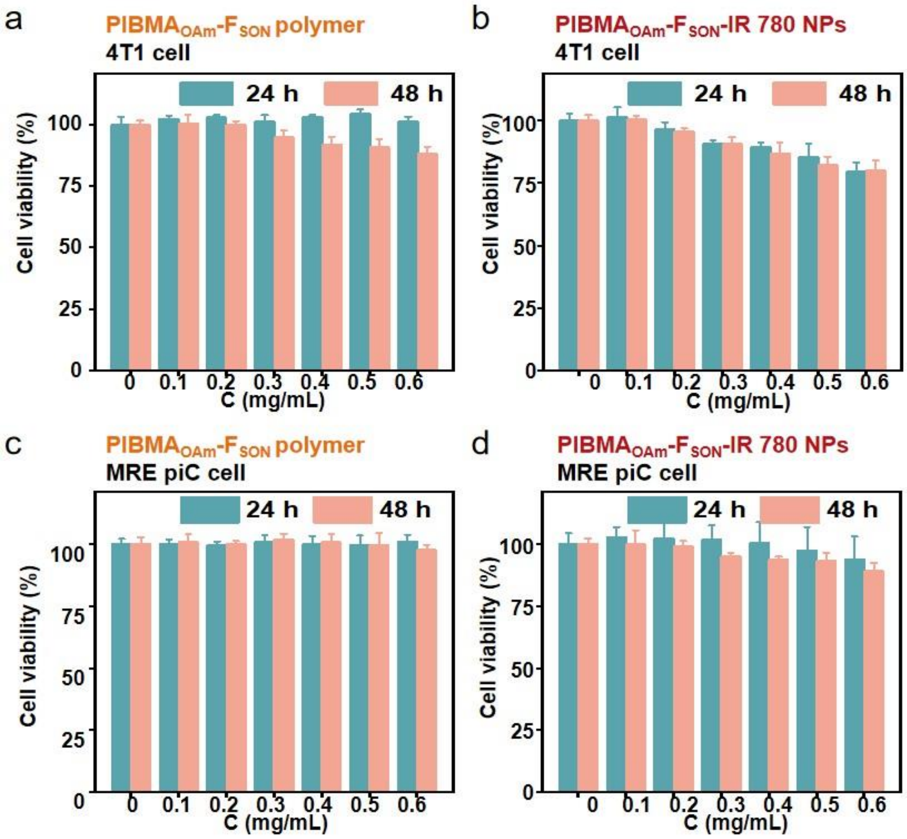

Figure S38. Cell viability of PIBMA$_{OAm}$-F$_{SON}$ (a and c) and PIBMA$_{OAm}$-F$_{SON}$-IR780 NPs (b and d) on the 4T1 cell line (a and b) and MRE piC cell line (c and d) at different concentrations after incubation for 24 h and 48 h, respectively.

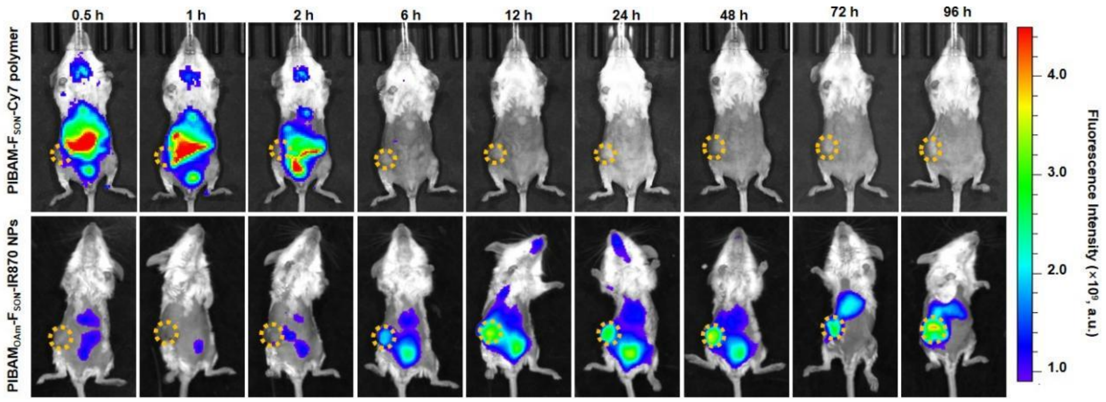

Figure S39. *In vivo* fluorescence imaging of 4T1 xenograft tumor model in BALB/c mice post intravenous (i.v.) injection with PIBMA-F$_{SON}$-Cy7 polymer or PIBMA$_{OAm}$-F$_{SON}$-IR780 NPs at different times. (Supine position) The tumor area was circled with yellow dot lines.

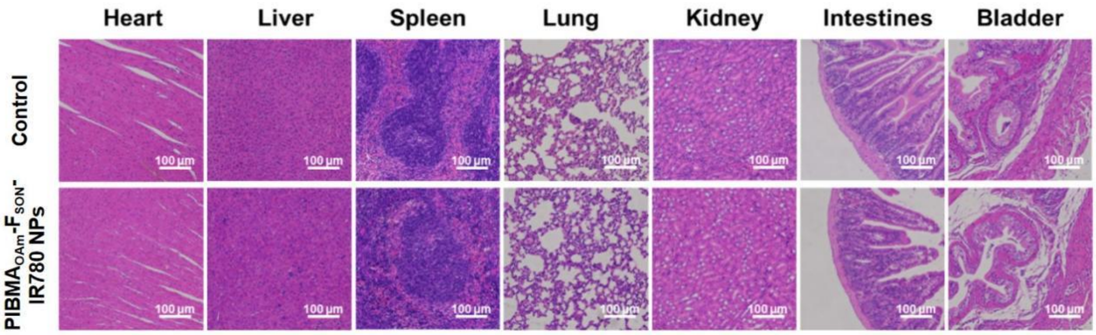

Figure S40. H&E-stained slices of the six organs including heart, liver, bladder, spleen, lung, and kidney from mice treated with PIBMA$_{OAm}$-F$_{SON}$-IR780 NPs.

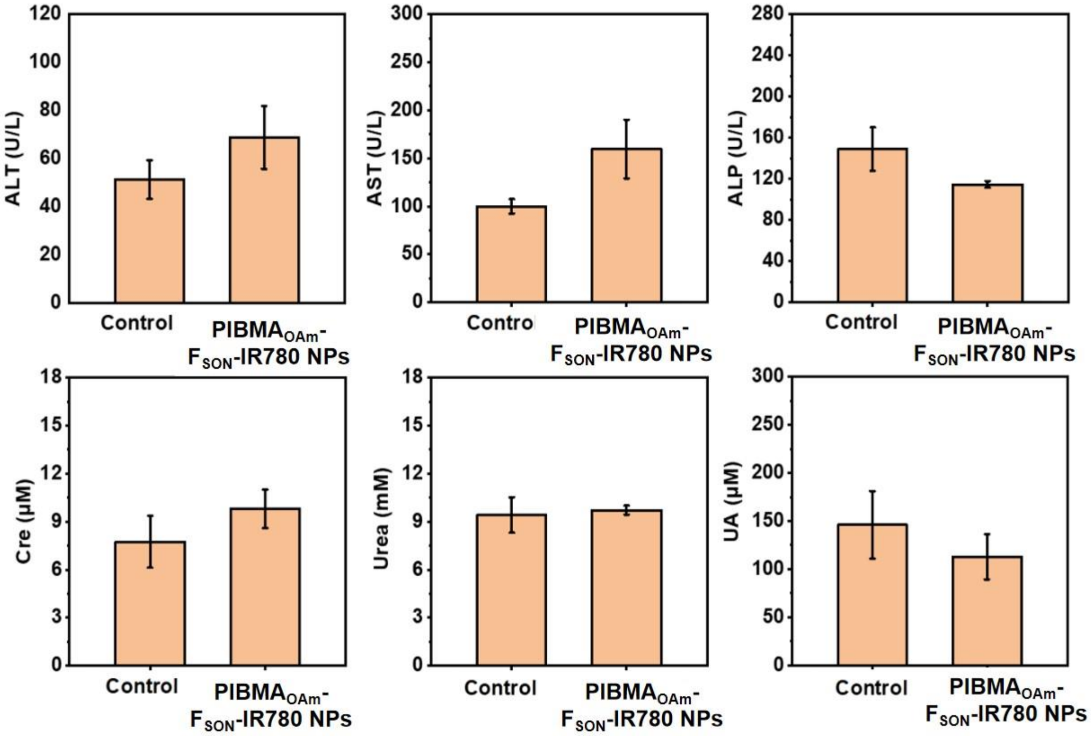

Figure S41. Serum biochemistry assay data to evaluate the ALT, AST, ALP, Cre, Urea and UA levels in mice receiving i.v. injection of PIBMA$_{OAm}$-F$_{SON}$-IR780 NPs. Data are presented as mean ± SD (n = 3).

No significant changes in the blood level of such markers were observed at 1-week post-injection, indicative of no renal toxicity (Figure S41). Changes in liver function were observed after injection of PIBMA$_{OAm}$-F$_{SON}$-IR780 NPs, because most of PIBMA$_{OAm}$-F$_{SON}$-IR780 NPs were taken up by the liver in the blood circulation, but these liver function values were within normal ranges.

3. References

[1] Instruments, Malvern, Tech note: Zeta potential - an introduction in 30 minutes. **2005**, 1.

[2] Routkevitch, D.; Sudhakar, D.; Conge, M.; Varanasi, M.; Tzeng, S. Y.; Wilson, D. R.; Green, J. J. Efficiency of Cytosolic Delivery with Poly (β-Amino Ester) Nanoparticles Is Dependent on the Effective p*K*a of the Polymer. *ACS Biomater. Sci. Eng.* **2020**, *6*, 3411–3421.

[3] Wilson, D. R.; Rui, Y.; Siddiq, K.; Routkevitch, D.; Green, J. J.Differentially Branched ester Amine Quadpolymers with Amphiphilic and pH-sensitive Properties for Efficient Plasmid DNA Delivery. *Mol.Pharmaceutics*, **2019**, *16,* 655−668.

[4] Hausig F.; Sobotta F. H.; Richter F.; Harz D. O.; Traeger A.; Brendel J. C. Correlation between Protonation of Tailor-Made Polypiperazines and Endosomal Escape for Cytosolic Protein Delivery. *ACS Appl. Mater. Interfaces,* **2021**, *13,* 35233-35247.
